# Enhanced viral infectivity and reduced interferon production are associated with high pathogenicity for influenza viruses

**DOI:** 10.1371/journal.pcbi.1010886

**Published:** 2023-02-09

**Authors:** Ke Li, James M. McCaw, Pengxing Cao

**Affiliations:** 1 School of Mathematics and Statistics, The University of Melbourne, Parkville, VIC, Australia; 2 Peter Doherty Institute for Infection and Immunity, The Royal Melbourne Hospital and The University of Melbourne, Parkville, VIC, Australia; 3 Melbourne School of Population and Global Health, The University of Melbourne, Parkville, VIC, Australia; Emory University, UNITED STATES

## Abstract

Epidemiological and clinical evidence indicates that humans infected with the 1918 pandemic H1N1 influenza virus and highly pathogenic avian H5N1 influenza viruses often displayed severe lung pathology. High viral load and extensive infiltration of macrophages are the hallmarks of highly pathogenic (HP) influenza viral infections. However, it remains unclear what biological mechanisms primarily determine the observed difference in the kinetics of viral load and macrophages between HP and low pathogenic (LP) viral infections, and how the mechanistic differences are associated with viral pathogenicity. In this study, we develop a mathematical model of viral dynamics that includes the dynamics of different macrophage populations and interferon. We fit the model to *in vivo* kinetic data of viral load and macrophage level from BALB/c mice infected with an HP or LP strain of H1N1/H5N1 virus to estimate model parameters using Bayesian inference. Our primary finding is that HP viruses have a higher viral infection rate, a lower interferon production rate and a lower macrophage recruitment rate compared to LP viruses, which are strongly associated with more severe tissue damage (quantified by a higher percentage of epithelial cell loss). We also quantify the relative contribution of macrophages to viral clearance and find that macrophages do not play a dominant role in the direct clearance of free viruses although their role in mediating immune responses such as interferon production is crucial. Our work provides new insight into the mechanisms that convey the observed difference in viral and macrophage kinetics between HP and LP infections and establishes an improved model-fitting framework to enhance the analysis of new data on viral pathogenicity.

## Author summary

Infections with highly pathogenic (HP) influenza virus (e.g., the 1918 pandemic H1N1 virus) often lead to serious morbidity and mortality. HP influenza virus infection is characterised by rapid viral growth, high viral load and excessive infiltration of macrophages to the lungs. Despite extensive study, we do not yet fully understand what biological processes lead to the observed viral and macrophage dynamics and therefore viral pathogenicity. Experimental studies have previously suggested that both viral factors (e.g., viral proteins) and host factors (e.g., the host immune response) play a role to enhance viral pathogenicity. Here, we utilise *in vivo* kinetic data of viral load and macrophages and fit a viral dynamic model to the data to estimate model parameters. Our model allows us to explore the biological mechanisms that contribute to the different viral and macrophage dynamics between HP and low pathogenic (LP) infections. This study improves our understanding of the role of interferon in distinguishing immunodynamics between HP and LP infections. Our findings may contribute to the development of next-generation treatments which rely upon an understanding of the different host immunological responses to HP influenza viruses.

## Introduction

Influenza is a contagious respiratory disease caused by influenza virus and remains a major public concern [1]. Infections associated with the highly pathogenic (HP) 1918 pandemic H1N1 virus and highly pathogenic avian H5N1 virus often display severe lung pathology, causing fatal infection outcomes in humans [2–4]. Animal models have been used to understand the mechanisms of viral pathogenicity [5–8]. High pathogenicity of viruses is often determined by pathogenic outcomes (e.g., the clinical outcomes of infection) in humans [2, 3, 9, 10]. The pathogenicity of influenza virus is not only associated with viral factors (e.g., viral HA protein), but is also influenced by host factors (e.g., the strength of inflammatory response), as reviewed in [11]. For example, although macrophages are important to orchestrate the host immune response, they are also implicated to damage cells through secreted inflammatory cytokines [12–15]. Some HP viruses can use macrophages as target cells and produce new virus from infected macrophages, altering the antiviral role of macrophages and contributing to viral infection [12, 16, 17]. Perrone et al. compared the outcome of infections with HP and LP strains of two influenza A viruses (i.e., the 1918 pandemic H1N1 virus and an H5N1 virus) in mice and showed that high-pathogenic viruses exhibited a significantly higher viral load as early as one day post-infection and a higher number of macrophages in the lungs [18]. However, the temporal dynamics of these viral or host factors, and so the major determinants of the observed differences in viral and macrophage kinetics between HP and LP, remain poorly understood.

Mathematical models have been used to study infection dynamics of influenza virus and its interactions with the host immune response (reviewed in [19]). To explore the potential mechanism(s) leading to the observed difference in viral loads and macrophages between HP and LP infections in [18], Pawelek et al. [20] fitted a mathematical model to the viral load and macrophage data and found that a higher activation rate of macrophages and an active production of viruses by macrophages infected with HP viruses are key drivers leading to higher viral loads and higher macrophage numbers [20]. More recently, Ackerman et al. [21] fitted a set of mathematical models with different hypothesised mechanisms—leading to distinct immunoregulatory behaviours (e.g., macrophage dynamics)—to strain-specific immunological data from [22]. They identified that different interferon production rates are the main causes of variance between infection outcomes in mice infected with low-pathogenic H1N1 or high-pathogenic H5N1 influenza viruses. The two modelling studies provided useful insights into the mechanisms of high pathogenicity and set a framework for assessing other potential mechanisms.

The two modelling studies [20, 21] also left aspects for improvement, both biologically and methodologically. Interferon-mediated immune response, which has been shown to be important for reducing epithelial loss [23], was not considered in [20]. Although the study by Ackerman et al. modelled interferon, they did not compare HP and LP viruses of the same type (rather they compared H5N1 HP vs. H1N1 LP) [21]. Through this study, we aim to identify viral and host factors that determine the observed difference in viral load and macrophage kinetics between HP and LP viruses from the same phenotype. Besides, both modelling studies used least-squares methods to provide point estimates to model parameters, which may not accurately quantify the uncertainty of estimated parameters and therefore limits our ability to draw reliable conclusions based on parameter estimates [24]. Recent advances in Bayesian statistical inference provide an improved framework for parameter estimation and quantification of uncertainty [25]. We would like to address the above limitations by building an improved framework to study the mechanisms for viral pathogenicity.

In this study, we develop a novel mathematical model which includes macrophage dynamics (i.e., resting, *M*_1_ and *M*_2_ macrophages), interferon-mediated immune responses and essential interactions between macrophage and virus. Under a Bayesian statistical framework, we fit the model to available *in vivo* kinetic data for both virus and macrophage populations of both highly pathogenic and paired low pathogenic strains of H1N1 or H5N1 viruses. We use the data-calibrated model to generate and compare a set of metrics that have been used as surrogates for viral pathogenicity [26, 27]. We identify the important role of interferon in distinguishing immunodynamics and the antiviral role of macrophages between HP and LP infections. We also demonstrate that our model reliably captures observed pathogenic behaviours (e.g., the severity of epithelium loss) and provides a quantitative estimation of the percentage of damaged cells during HP and LP infections.

## Results

### Severe tissue damage in HP infection

**Fig 1:**
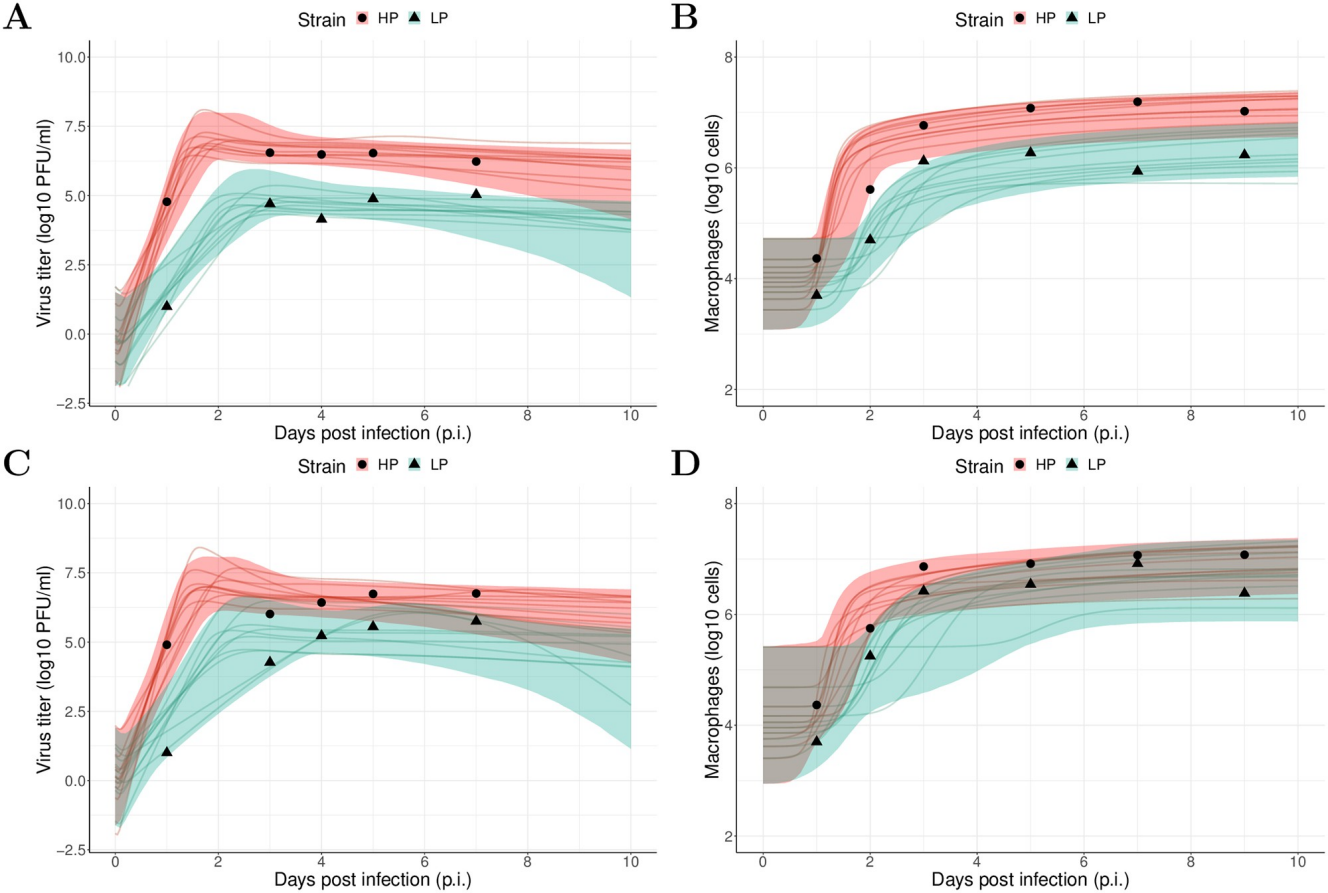
Results of model fitting for virological and macrophage data. Data are presented by solid circles for HP and solid triangles for LP strains. As mentioned in the Materials and methods, the data were adopted from [18], and macrophage data represented the sum of all three subpopulations of macrophages (i.e., *M*_*R*_ + *M*_1_ + *M*_2_). We performed 6000 model simulations based on 6000 posterior samples from the posterior distributions of estimated parameters (see S1 Fig for the H1N1 viurses and S2 Fig for the H5N1 viruses). (A, B) show a 95% prediction interval (shaded area) of viral load and macrophage for HP (red) and LP (green) strains of the H1N1 viruses, respectively. Solid lines are illustrative viral and macrophage trajectories. (C, D) show the data and model predictions of viral load and macrophage dynamics for HP and LP strains of the H5N1 viruses, respectively.

We fit our model to both viral load and macrophage data of HP and LP strains simultaneously under a Bayesian framework (the details of the model and statistical implementation, and full diagnostics on the statistical procedures are provided in the Materials and methods). Model fitting results for H1N1 viruses are given in Fig 1. Our model successfully captures the trends of both viral load (Fig 1A) and macrophage number (Fig 1B) for both the HP and LP strains of H1N1 viruses, and a low level of overlapping of the 95% prediction interval (PI, shaded area) between HP and LP suggests that the quantitative differences in viral load and macrophage are primarily due to different parameter values associated with different strains rather than measurement errors. In terms of goodness-of-fit, similar fitting results are observed for infection with the HP and LP strains of H5N1 viruses (Fig 1C and 1D), such that our model also successfully captures the trends of viral load and macrophage dynamics for the H5N1 viruses. Note that there is a larger degree of overlap in the 95% prediction intervals for the modelled macrophage kinetics for H5N1 HP and LP viruses (Fig 1D) compared to those for H1N1 HP and LP viruses (Fig 1B). This is consistent with the fact that the macrophage data for the two H5N1 strains are more similar than for the two H1N1 strains.

**Fig 2:**
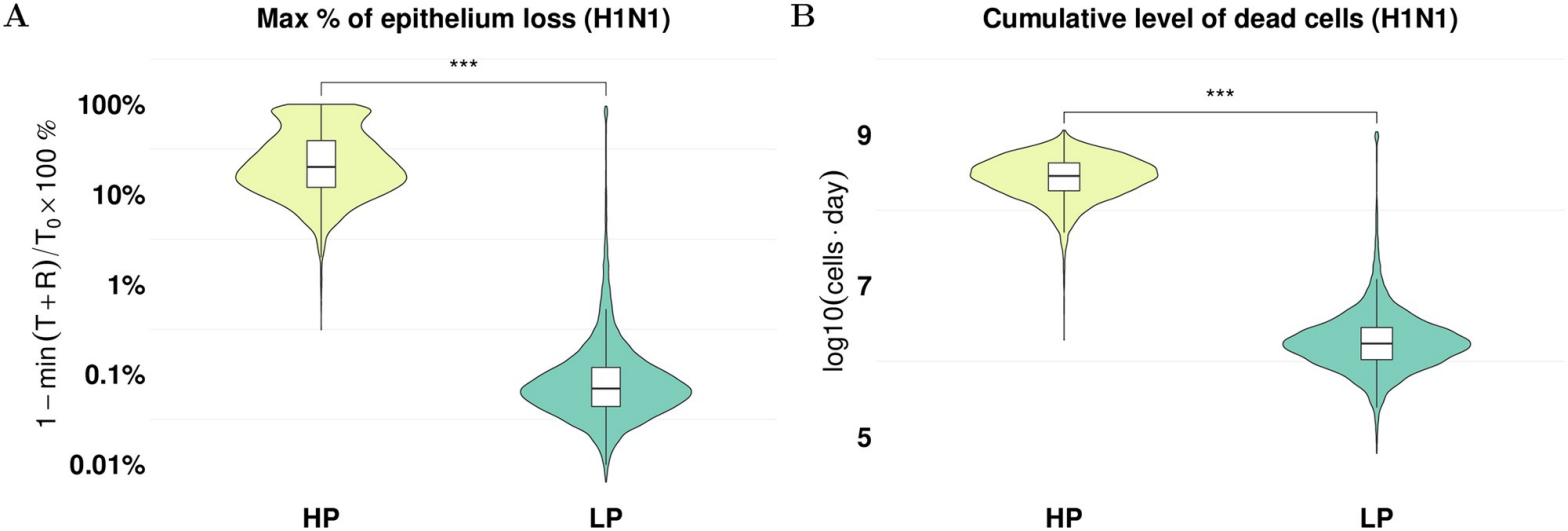
Prediction of tissue damage for H1N1 viruses. The violin plots (coloured) and boxplots (white) give the density and the median and extrema of the predicted quantity. (A) model prediction of the maximal epithelium loss for the HP (yellow) and LP (green) strains. (B) model prediction of the cumulative level of dead cells during the infection for both strains. ***p<0.001. For calculation formulas see Eqs 11 and 12 in the Materials and methods. All estimations are computed using 6000 posterior samples from model fitting. The estimations for the H5N1 viruses are given in S3 Fig.

Using the calibrated model, we then calculate the maximal percentage of epithelium loss (defined by Eq 11 in Materials and methods) and the cumulative dead cells (Eq 12 in Materials and methods) during infection which is difficult to measure experimentally but are important indicators of infection severity. For H1N1 viruses, our model predicts a much larger percentage of epithelial cells (median value 20.06%, 95% predict interval (PI): [4.39%, 94.19%]) are damaged during the HP infection compared to that in the LP infection (median value 0.07%, 95% PI: [0.02%, 1.08%]), as shown in Fig 2A. Similarly, for the cumulative number of dead cells shown in Fig 2B, we observe that there is a high cumulation of dead cells (median log_10_(*AUC*_*D*_) 8.5, 95% PI: [7.7, 8.9]) in the HP infection whereas the cumulation of dead cells is low in LP infection (median log_10_(*AUC*_*D*_) 6.2, 95% PI: [5.5, 7.1]).

### A high viral infectivity and a low interferon production contribute to severe tissue damage in HP infection

**Fig 3:**
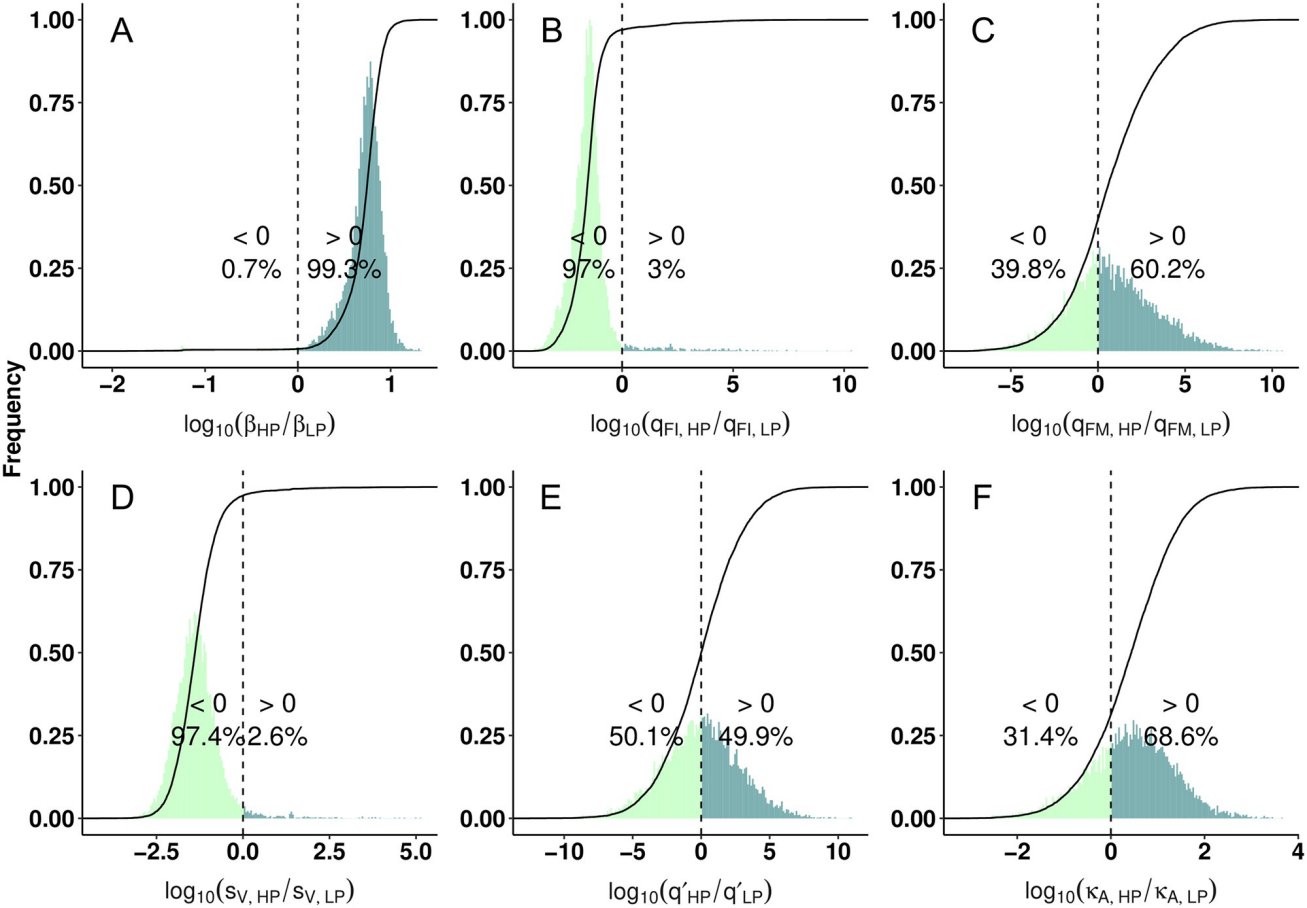
Comparison of estimated model parameters between HP and LP strains of the H1N1 viruses. Histograms show the frequency of the ratios of estimated HP parameters over paired LP model parameters and are normalised to [0, 1]. The ratios are presented by distributions of 6000 samples because they are generated by 6000 posterior parameter values. The cumulative density functions (CDFs) are given by the solid lines, and the dashed lines indicate ratios = 0. All ratios are log10-scaled, such that ratios > 0 (dark green) suggest greater values of the HP parameters. Fig (A, B, C) show the ratios of viral infectivity, and interferon production rate from infected cells and activated macrophages, respectively. Fig (D, E, F) show the ratios of infection-induced macrophage recruitment rate, macrophage-mediated virus clearance rate and antibody neutralisation rate, respectively. The model parameter comparison for the H5N1 viruses is given in S4 Fig.

Given the significant difference in tissue damage between HP and LP viruses, we now investigate the underlying biological processes that are responsible for the difference. We examine six biological model parameters that may convey the difference between HP and LP virus (i.e., the six parameters assumed to be different between HP and LP in model fitting. see Materials and methods for details). To make a direct comparison, we present the ratio of HP’s estimate to LP’s estimate for each parameter in Fig 3 (note that for each parameter there are 6000 ratio values calculated by 6000 paired HP and LP posterior values and thus we show the distribution of the 6000 ratio values in the figure). We observe that for H1N1 the HP strain has a significantly higher viral infectivity *β* (99.3% of the ratio samples are greater than 1 as indicated by dark green. Fig 3B and 3C compare the interferon production rate from infected cells, *q*_*FI*_, and from activated macrophages, *q*_*FM*_, respectively. We find that although the HP strain has a decreased *q*_*FI*_, such that 97% ratio samples are lower than 1 (indicated by light green), there is no strong evidence to indicate a difference in *q*_*FM*_, i.e., approximately half of the posterior estimates for ratios are below 1 (39.8%) and approximately half are above 1 (60.2%). The results demonstrate that the HP virus is more capable of infecting susceptible cells and reducing interferon response from infected cells. The results are supported by a variety of experimental studies where enhanced infection and replication rates [28, 29] and attenuated interferon production rates [3, 9, 12, 13, 30–34] are evidenced as possible explanations to high viral pathogenicity.

Fig 3D shows that the rate of infection-induced macrophage recruitment *s*_*V*_ is lower for the HP strain (97.4% of the ratio samples are less than 1), suggesting that a high recruitment rate is not the cause for the observed high level of macrophages during the HP infection seen in Fig 2. Instead, our model result indicates that the high level of macrophages is due to a higher number of infected cells which activate more macrophages. A similar finding was shown by Shoemaker et al. who found that a strong inflammation-associated gene expression occurs once a threshold virus titer is exceeded, demonstrating a strong dependency between the extent of the inflammation and the level of virus titer [22].

**Fig 4:**
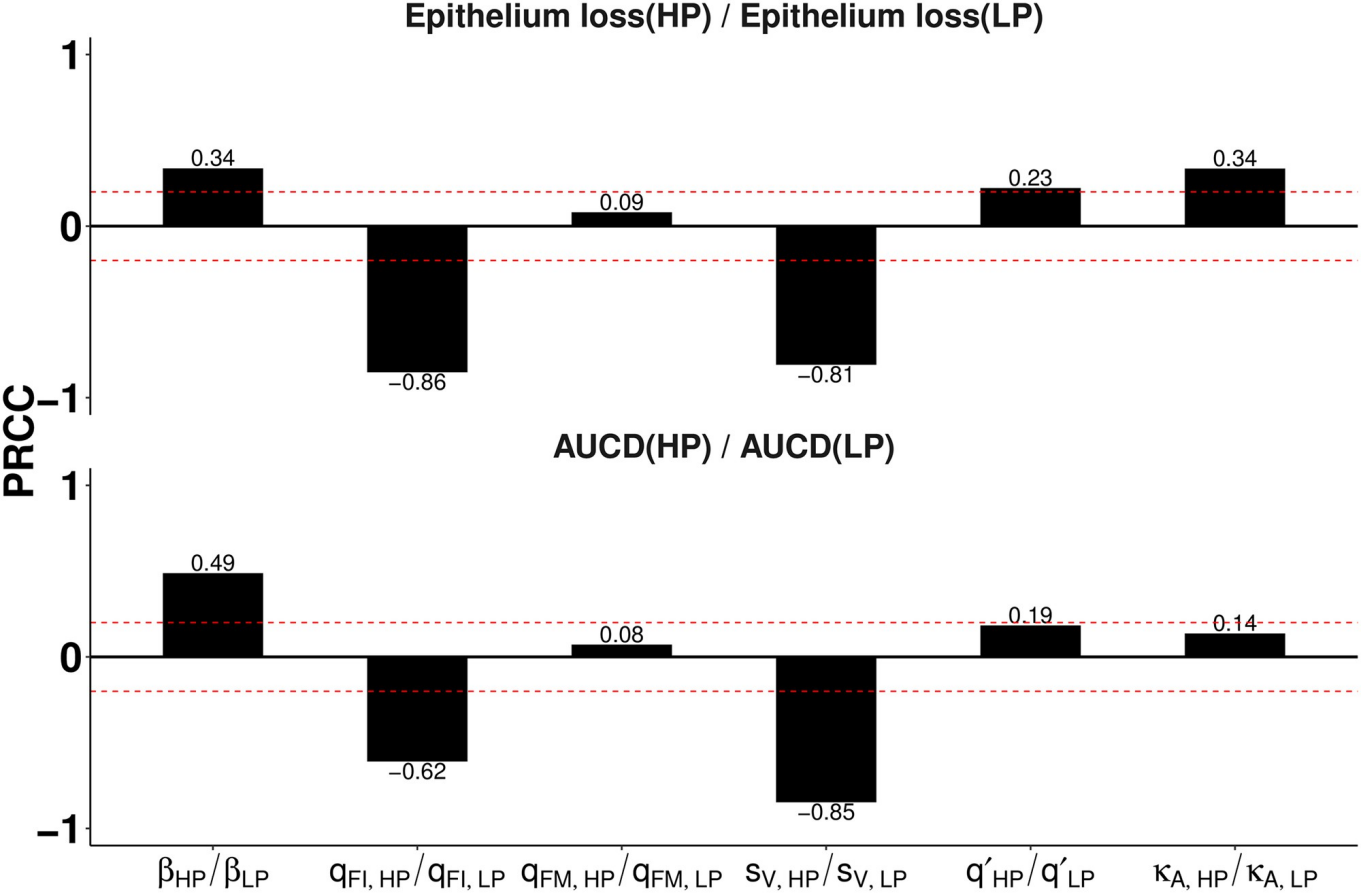
Correlations between estimated model parameters and tissue damage. Partial rank correlation coefficients (PRCC) are calculated with respect to (A) the ratio of max epithelium loss between HP and LP strains, and (B) the ratio of the cumulative dead cells between HP and LP strains of H1N1 viruses. The two red dashed lines represent the statistically insignificant values of PRCC. Calculations are based upon 6000 posterior samples from model fitting. PRCC analysis for H5N1 viruses is given in S5 Fig.

We further examine how the difference in estimated parameters between HP and LP is associated with the different estimated levels of tissue damage shown in Fig 2. We calculate the Partial Rank Correlation Coefficients (PRCCs) between the ratio of estimated parameters and the ratio of epithelium loss between HP and LP strain. We find that the interferon production rate *q*_*FI*_ and infection-induced macrophage recruitment rate *s*_*V*_ are the two leading factors determining the maximum epithelium loss (Fig 4A) and they are negatively correlated with the maximum epithelium loss (PRCC = −0.86 and −0.81 respectively). Analysing the cumulative number of dead cells using the same method, we also find that *q*_*FI*_ and *s*_*V*_ are the two leading parameters driving the difference in the cumulative number of dead cells (Fig 4B), with again negative correlations (PRCC = −0.62 and −0.85). By contrast, the ratio of viral infectivity *β* has a relatively small effect on the ratio of maximum percentage epithelium loss and on the ratio of the cumulative number of dead cells. Our results suggest a critical role of interferon in protecting against epithelium loss and tissue damage, given *q*_*FI*_ directly determines the rate of interferon production and *s*_*V*_ has an indirect contribution via generating more *M*_1_ macrophages that directly promotes the rate of interferon production (see model Eq 8 in the Materials and methods). The results are consistent with the earlier finding that interferon can retain a large healthy epithelial cell pool for viral re-infections [23] and are supported by Ackerman et al. [21] who found that different interferon production rates are the main causes of variance between infection outcomes in mice infected with low-pathogenic H1N1 and with high-pathogenic H5N1 influenza viruses.

### The role of macrophages to viral clearance

**Fig 5:**
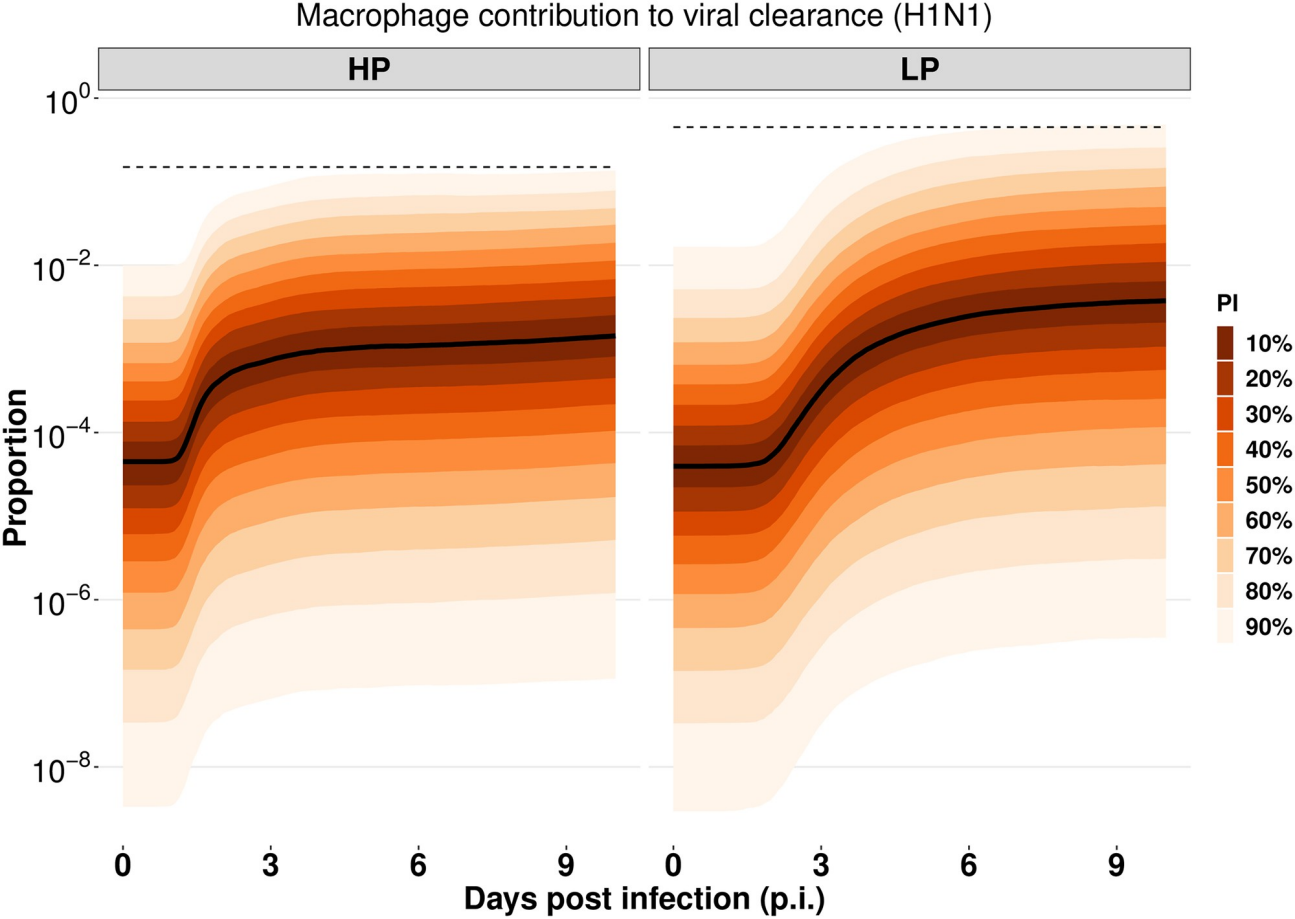
The relative contribution of macrophages to viral clearance in the HP and LP strains of the H1N1 viruses. The prediction interval (PI) is calculated based on the 6000 posterior samples from model fitting. The median trajectory is indicated by the black curve. The predictions for the H5N1 viruses are given in S6 Fig.

As described in the introduction, the reduced antiviral effect of macrophages may contribute to viral pathogenicity. We here analyse the role of macrophages to viral clearance in both HP and LP infections. In our model, viruses are cleared through three ways: natural decay, macrophage phagocytosis and antibody neutralisation. We use the (Eq 13 in the Materials and methods) to quantify the contribution of macrophage phagocytosis over the period of infection by a fractional value (e.g., 0 means no contribution and 0.5 means 50% of viral clearance rate is due to macrophage phagocytosis). The prediction interval (PI) can be used to quantify the uncertainty of the contribution fraction. As shown in Fig 5, for the H1N1 virus, 90% of model predicted fractions of the contribution of macrophages to viral clearance (indicated by the 90% PI) are below 0.15 for HP and are below 0.45 for LP (indicated by the dashed lines). The upper bounds of the contribution fractions drop significantly for the high-confidence range of model predictions, e.g., 60% of model predicted fractions (indicated by the 20% PI) are less than 0.5% for HP and less than 1.1% for LP. The results indicate the antiviral effect of macrophages is likely to be limited in both LP and HP infections. We also compare the relative contribution of macrophages in the HP and LP H5N1 viruses (S6 Fig) and find a similar result as in the H1N1 viruses. The result suggests that although macrophages are critical to orchestrating the host immune responses, e.g., initiating and resolving pulmonary inflammation, they are unlikely to be the dominant mechanism to clear free viruses.

### Predicting the effective ways to reduce tissue damage

**Fig 6:**
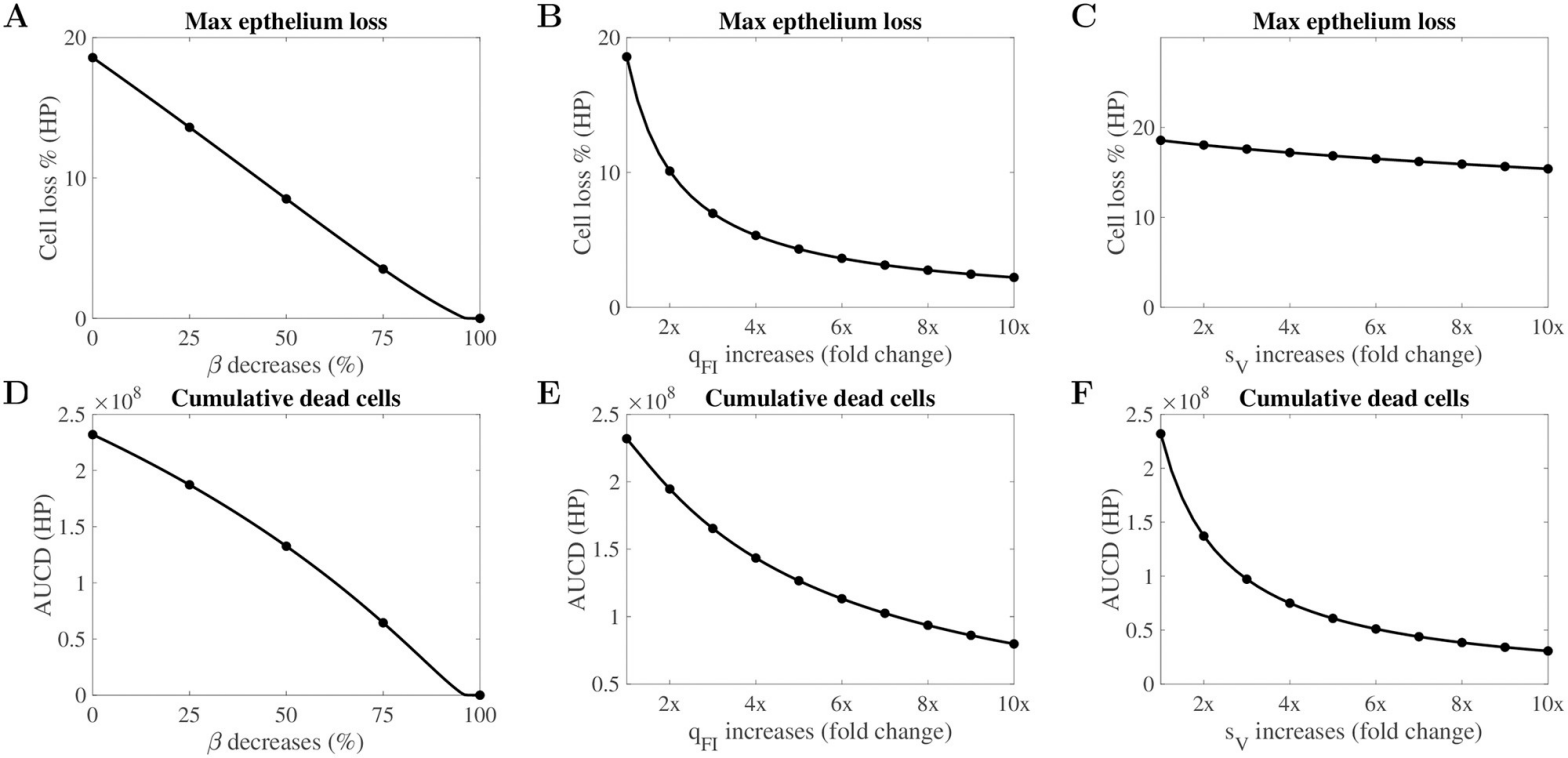
Parameters driving tissue damage for the HP H1N1 virus. Fig (A, B, C) give the sensitivity analyses of the impact of *β*, *q*_*FI*_ and *s*_*V*_ on maximal epithelium loss. Fig (D, E, F) show the impact of the same three model parameters on the cumulative dead cells. The baseline values for the parameters were chosen so that the median value of the maximal percentage of tissue damage (panels A, B, C) or the cumulative dead cells (panels D, E, F) corresponds to the value from our main analysis (Fig 2A and 2B respectively). Parameters driving tissue damage for the LP H1N1 virus are given in S7 Fig.

Since *β*, *q*_*FI*_ and *s*_*V*_ have been shown to be the leading factors correlated with the tissue damage (Fig 4), modulating those parameters may provide effective ways to reduce the tissue damage. Fig 6A, 6B and 6C show the impact of varying *β*, *q*_*FI*_ and *s*_*V*_ on the maximal percentage of epithelium loss for HP infection, respectively. We find that while decreasing *β* (Fig 6A) or increasing the interferon production rate *q*_*FI*_ (Fig 6B) can both significantly prevent epithelium loss in infection, the marginal effect on reducing epithelium loss by increasing *q*_*FI*_ decreases, such that there exists a non-linear relationship between epithelium loss and the interferon production rate. For example, doubling the production rate halves the epithelium loss, (i.e., epithelium loss is reduced from 20% to 10%). Reducing 90% of cell loss, however, requires a factor of 10 increase in *q*_*FI*_. We also note that an enhanced infection-induced macrophage recruitment rate *s*_*V*_ has almost no influence on epithelium loss for HP infection (Fig 6C). Fig 6D, 6E and 6F show the dependency of cumulative dead cells upon *β*, *q*_*FI*_ and *s*_*V*_ for HP infection, respectively. We find the cumulative dead cell number is sensitive to all three parameters. The results suggest that minimising viral infectivity and boosting interferon production can provide an effective way to reduce the maximum percentage of epithelium loss during infection. The results also imply that increasing the macrophage recruitment rate can reduce the cumulative number of dead cells, providing a possible pathway to reduce inflammation. For comparison, we also compute the dependency of tissue damage on the three parameters for the LP virus (S7 Fig) with similar findings. Note that although the actual magnitude of epithelium loss is minor for the LP virus, the percentage change is comparable between HP and LP strains.

## Discussion

In this work, we identified biological mechanisms that are associated with high pathogenicity of *in vivo* H1N1 and H5N1 infections by fitting a viral dynamic model to experimental data under a Bayesian framework. Our findings support and contribute to the current knowledge that is relevant to two frequently studied experimental explanations on the drivers of high pathogenicity for influenza viruses (i.e., higher viral infectivity and a reduced interferon response). Estimated marginal posterior densities of model parameters demonstrate that HP viruses have enhanced viral infection rates (i.e., higher *β*) and reduced interferon production rates (i.e., lower *q*_*FI*_) compared to LP viruses. Our estimation results also explain the difference in viral and macrophage kinetics between HP and LP infections. As shown by previous studies [23, 35, 36], a higher viral infection rate leads to faster viral growth and an attenuated interferon production leads to a higher peak viral loads.

Our work quantified the difference in tissue damage between HP and LP infections. We predicted a larger percentage of epithelium loss and a higher cumulative dead cells are caused in HP infections (Fig 2 for H1N1 and S3 Fig for H5N1). We note the high level of uncertainty in the predicted maximum percentage of epithelial cell loss for the HP H1N1 virus. This is a result of a large 95% credible interval of the interferon production rate *q*_*FI*_ (spanning over two orders of magnitude as shown in S1(B) Fig) which has been shown to be highly correlated with the maximum percentage of epithelial cell loss.

Our model predictions—a high percentage of epithelium loss and a high cumulative dead cells in HP infection—are supported by clinical evidence. Severe destruction of lung tissue [2] and severe tissue consolidation with unique destruction of the lung architecture [2, 37] have been seen in patients infected with HP influenza viruses, leading to lung pathology [28, 30, 38–40]. The severity of tissue damage also resulted in different mechanisms of viral resolution. While target cell depletion remains a mechanism to limit viral replication in HP infections, a timely and strong activation of immune response explains viral resolution in LP infections (see S8 Fig for the H1N1 viruses and S9 Fig for the H5N1 viruses). As shown by Cao and McCaw, the mechanisms for viral control can strongly influence the predicted outcomes of antiviral treatments [41]. For example, different viral dynamics (e.g., long-last infection or chronic infection) were observed in response to an increasing drug efficacy when target cell depletion is a mechanism for viral resolution. In contrast, a consistent viral behaviour (i.e., an early clearance and a shorter infection) was observed when drug efficacy increased in an immune response-driven viral resolution model. Therefore, the analysis of the influence of antiviral treatment on HP and LP infections is a promising future direction based on our work.

Using a Bayesian statistical method, our modelling work demonstrated that the high virulence of H1N1 and H5N1 viruses is associated with enhanced viral infectivity and attenuated interferon responses, supporting previous experimental studies [32, 42–44]. Although our work identified HP and LP viruses differ in viral infectivity and interferon production rates, we cannot (and do not attempt to) rule out other possible mechanisms or drivers of high pathogenicity proposed in the literature. For example, the production of viruses by infected macrophages could be an important factor influencing viral pathogenicity [17], although there is conflicting evidence on whether macrophages can be productively infected by influenza virus [15, 16, 45]. The abortive or productive infection of macrophages may also be strain-dependent and/or macrophage-dependent (i.e., resident or monocyte-derived macrophages) [17]. Thus, we did not explicitly investigate this mechanism in our study.

Viral dynamical models are particularly useful in the quantification of modelled biological processes by fitting to experimental data [19]. In this work, we fit our model to both viral load and macrophage data to estimate model parameters. Using a simulation-estimation method, we showed that macrophage data provides invaluable information on parameter estimation, reducing the uncertainty of predicted time series of macrophages and improving the estimates of the recruitment rates of macrophages (i.e., *s*_*M*_ and *s*_*V*_). By contrast, viral load data alone are insufficient to reliably recover macrophage dynamics (see S3 Text). Macrophages have been shown to clear viruses by internalisation and lysosomal degradation [46, 47], but their relative contribution to viral clearance compared to other pathways has not been quantified. Our model predicted the contribution of macrophages to viral clearance (among all the modelled mechanisms for viral clearance) is small in both HP and LP infections of H1N1 (Fig 5) and H5N1 (S6 Fig) viruses, suggesting that macrophages do not play a dominant role in the direct clearance of free virions.

Our study has some limitations. Rather than explicitly modelling the dynamics of CD8^+^ T cells and antibodies [35, 48], we used Hill functions to capture their dynamics. We assumed the adaptive immune response dominates infected cell or viral clearance at day 5 post-infection regardless of macrophage dynamics, as shown in [49]. Macrophages, however, have been shown to act as antigen-presenting cells and mediate the activation of different arms of adaptive immunity. For example, *M*_1_ type macrophages help to activate the cellular adaptive immune response whereas *M*_2_ type macrophages contribute to the activation of humoral adaptive immunity [50, 51]. Extension of the model to include the interactions between different populations of macrophages and adaptive immunity is important but requires additional data on the adaptive immune response for both HP and LP, which are not immediately available in the literature. Another limitation is that we did not estimate conversion rates between different populations of macrophages, such as *k*_1_ and *k*_2_, due to a lack of detailed macrophage kinetic data. As a result, the kinetics for each specific macrophage population could not be calibrated against data. The interactions among macrophage populations, e.g., the rate of conversion from one type to another, could be an important factor to understand influenza disease severity. In future work, our model can be used to estimate the relevant parameters and predict detailed macrophage dynamics given the availability of data from different macrophage populations.

## Materials and methods

### Mathematical model

**Fig 7:**
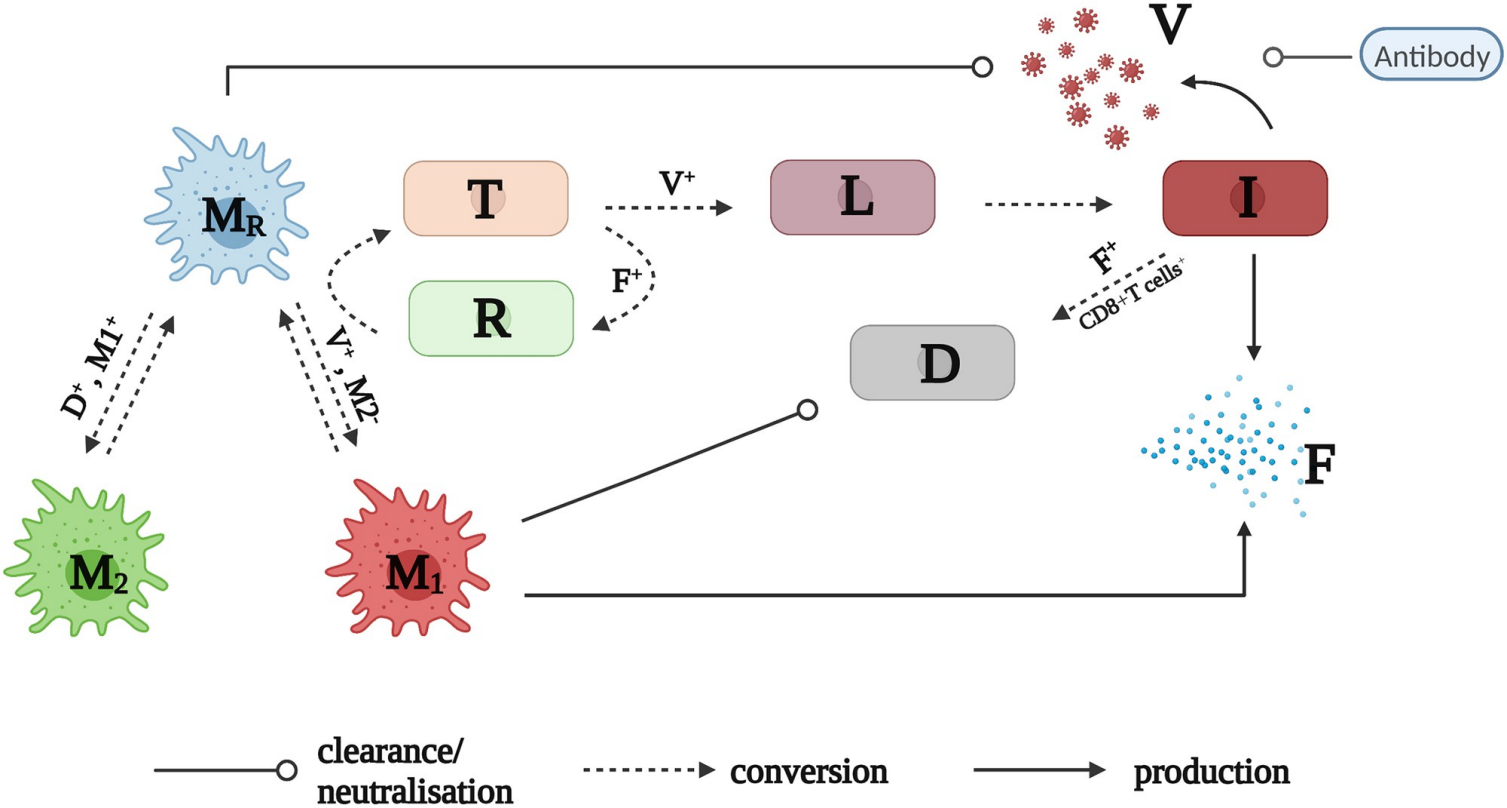
A model diagram of immune response to influenza viral infection. A detailed model (Eqs 1–10)) description is given in Materials and Methods. Plus (+) superscript indicates the promotion of a biological process, and minus (−) superscript means the inhibition of a process. In brief, influenza virus (*V*) turns susceptible epithelium cells (*T*) into eclipse-phase infected cells (*L*) which in turn, become infected cells (I) that actively produce new viruses. The virus also infects resting macrophages (*M*_*R*_) and turns them into pro-inflammatory macrophages (*M*_1_). Viruses are cleared through the *M*_*R*_ macrophage ingestion and antibody neutralisation. Infected cells (*I*) and *M*_1_ macrophages produce interferons (*F*) that turns susceptible cells (*T*) into refractory cells (*R*). The refractory cells (*R*) lose protection and turn back to *T*. Infected cells (*I*) are killed and become dead cells (*D*) through interferons- and CD8^+^ T cells-mediated clearance. *M*_1_ macrophages clear dead, which facilitates the conversion of *M*_*R*_ to anti-inflammatory *M*_2_ macrophages. Both activated *M*_1_ and *M*_2_ macrophages convert back to *M*_*R*_ macrophages at certain rates. For clarity, flows depicting the natural decay of activated macrophages (*M*_1_ and *M*_2_), virus (*V*) and interferons (*F*), and the replenishment of resting macrophages (*M*_*R*_) and target cells (*T*) are not shown in the diagram. The figure was created with BioRender.com.

In this study, we incorporated a dynamic model of macrophages into a viral dynamic model. The model explicitly considered the conversion among different populations of macrophages, essential interactions between virus and macrophages, and different arms of immune responses. The model is described by a set of ordinary differential equations, and a model diagram is given in Fig 7.

Eqs 1–3 describe the detailed macrophage dynamics. In the absence of viral infection, we assume all macrophages are resting macrophages (*M*_*R*_), and *M*_*R*_ is assumed to have a constant supplementary rate and decay rate at *s*_*M*_ and *δ*_*MR*_ per day, respectively. Thus, the number of macrophages is stable at homeostasis, such as $M_{R}^{*}=s_{M}/\delta_{MR}$ in a disease-free condition. In the presence of viral infection, influenza virus acting as a perturbation to macrophage dynamics, activates *M*_*R*_ macrophages, turning them into pro-inflammatory macrophages *M*_1_ at a maximal rate *k*_1_. The activation is influenced by viral load (*V*/(*V* + *V*_50_)) and regulated by anti-inflammatory *M*_2_ macrophages (1/(1 + *αM*_2_)). We also assume the recruitment rate of *M*_1_ macrophages from blood to the site of infection (*s*_*V*_) is proportional to the level of infection, which is indicated by the number of infected cells (*I*). A similar linear term has been used elsewhere [52] where the recruitment rate of resident macrophages during infection was modelled as proportional to the number of infected and activated macrophages. Activated *M*_1_ macrophages convert back to the resting macrophages or decay at constant rate *k*_−1_ and *δ*_*MA*_ per day, respectively. The *M*_2_ macrophages regulate the activation of *M*_1_ macrophages to avoid excessive inflammatory response [53]. *M*_1_ macrophages phagocyte apoptotic and dead cells, producing regulatory cytokines (not explicitly modelled), which is represented by *M*_1_*D*/(*D* + *D*_50_). In the presence of these cytokines, resting macrophages *M*_*R*_ convert to *M*_2_ macrophages at a maximal rate *k*_2_. Activated *M*_2_ macrophages decay or convert back to the resting state at constant rates *δ*_*MA*_ and *k*_−2_, respectively. In terms of macrophage dynamics, pro-inflammatory cytokines such as interleukin-6 (IL-6) and tumour necrosis factor-alpha (TNF-*α*) are produced by activated *M*_1_ macrophages [54, 55]. Evidence from laboratory studies [56, 57] has demonstrated that the level of these pro-inflammatory cytokines typically increases in the early stages of influenza infection before gradually declining afterwards. This kinetic behaviour is consistent with the kinetics of *M*_1_ macrophages predicted by our model (see S10 Fig for the H1N1 viruses and S11 Fig for the H5N1 viruses). Furthermore, *M*_2_ macrophages produce anti-inflammatory cytokines such as IL-4 and IL-10, and time series data of IL-4 from [58] (in which mice were experimentally infected with influenza A/PR/8/34 H1N1 virus) demonstrate qualitatively similar kinetics to the *M*_2_ macrophages predicted by our model (i.e., an initial decrease followed by a recovery back to baseline, as shown in S10 Fig for the H1N1 viruses and S11 Fig for the H5N1 viruses).

Eqs 4–7 describe the interaction between viruses and epithelial cells, and between viruses and the host immune responses. In detail, epithelial cells (*T*) are infected by influenza virus (*V*) and become latent-state infected cells (*L*) which do not produce new viruses at an infectivity rate *βV* per day. The susceptible epithelial cells are protected and convert to refractory cells (*R*) in the presence of interferon (*F*) at a rate *ϕF* per day, and refractory cells convert back to susceptible cells at a rate *ξ*_*R*_. We also assume susceptible cells are replenished at a rate *g*_*T*_(*T* + *R*)(1 − (*T* + *L* + *I* + *R*)/*T*_0_), where *T*_0_ is the maximal number of epithelial cells that line the upper respiratory tract. Infected cells in the eclipse phase convert to infected cells (*I*) that actively produce viruses at a rate *ℓ* per day. Three mechanisms are considered for the clearance of infected cells (*I*), such as natural decay at a constant rate *δ*_*I*_ per day; interferon-mediated clearance at a rate *κ*_*F*_*F* per day, and CD8^+^ T cells mediated infected clearance at a rate $\kappa_{E}t^{4}/\left(t^{4}+t_{E}^{4}\right)$ per day. Note that we do not explicitly model the dynamics of CD8^+^ T cells. A Hill function is used to represent the activation of adaptive immunity, we set *t*_*E*_ as 5 so that CD8^+^ T cells only play a significant role after day 5 post-infection as shown in [49]. New viruses are produced by *I* at a rate *p*_*I*_*I* viruses per day. The decrease of viruses is either due to natural decay, macrophage-mediated phagocytosis or antibody neutralisation at a rate *δ*_*V*_, *q*′*M*_*R*_, and $\kappa_{A}t^{4}/\left(t^{4}+t_{A}^{4}\right)$ per day, respectively. Here, we assume that only resting macrophages directly contribute to viral clearance. Resting macrophages, *M*_*R*_, are able to recognise and engulf free virions upon infection, reducing free virus (*V*) [17]. The primary role of *M*_1_ macrophages—activated from resting macrophages—is to initiate the inflammatory response and induce interferon production, which directly destroys infected cells. The role of *M*_2_ macrophages is to regulate *M*_1_ macrophage activation [54, 55].

Eqs 8 and 9 describe one of the interferon dynamics and the dynamics of refractory cells. We assume Interferon (*F*) is produced either by infected cells (*I*) or macrophages (*M*_1_) at a rate *q*_*FI*_*I* or *q*_*FM*_*M*_1_ unit of interferons per day, respectively, and decay rate a rate *δ*_*F*_ per day. The dynamics of dead cells (*D*) are described by Eq 10. Cleared infected cells (*I*) become dead cells (*D*) through *δ*_*I*_*I*, *κ*_*F*_*F* and $\kappa_{E}t^{4}/\left(t^{4}+t_{E}^{4}\right)$, and dead cells are removed from the system either due to natural decay at a rate *δ*_*D*_ per day or engulfed by macrophages at a rate *κ*_*D*_*M*_1_ per day.

$$\begin{array}{cc} & \frac{dM_{R}}{dt}=s_{M}-\delta_{MR}M_{R}-K_{1}\left(V,M_{2}\right)M_{R}+k_{-1}M_{1}-K_{2}\left(D,M_{1}\right)M_{R}+k_{-2}M_{2}, \end{array}$$ (1)

$$\begin{array}{cc} & \frac{dM_{1}}{dt}=s_{V}I+K_{1}\left(V,M_{2}\right)M_{R}-k_{-1}M_{1}-\delta_{MA}M_{1}, \end{array}$$ (2)

$$\begin{array}{cc} & \frac{dM_{2}}{dt}=K_{2}\left(D,M_{1}\right)M_{R}-k_{-2}M_{2}-\delta_{MA}M_{2}, \end{array}$$ (3)

$$\begin{array}{cc} & \frac{dT}{dt}=g_{T}\left(T+R\right)(1-\frac{T+L+I+R}{T_{0}})-\beta TV-\phi FT+\xi_{R}R, \end{array}$$ (4)

$$\begin{array}{cc} & \frac{dL}{dt}=\beta TV-\ell L, \end{array}$$ (5)

$$\begin{array}{cc} & \frac{dI}{dt}=\ell L-\delta_{I}I-\kappa_{F}FI-\kappa_{E}\frac{t^{4}}{t^{4}+t_{E}^{4}}I, \end{array}$$ (6)

$$\begin{array}{cc} & \frac{dV}{dt}=p_{I}I-\delta_{V}V-q^{\prime }M_{R}V-\kappa_{A}\frac{t^{4}}{t^{4}+t_{A}^{4}}V, \end{array}$$ (7)

$$\begin{array}{cc} & \frac{dF}{dt}=q_{FI}I+q_{FM}M_{1}-\delta_{F}F, \end{array}$$ (8)

$$\begin{array}{cc} & \frac{dR}{dt}=\phi FT-\xi_{R}R, \end{array}$$ (9)

$$\begin{array}{cc} & \frac{dD}{dt}=\delta_{I}I+\kappa_{F}FI+\kappa_{E}\frac{t^{4}}{t^{4}+t_{E}^{4}}I-\kappa_{D}M_{1}D-\delta_{D}D, \end{array}$$ (10)

where $K_{1}\left(V,M_{2}\right)=k_{1}\frac{V}{V+V_{50}}\frac{1}{1+\alpha M_{2}}$ and $K_{2}\left(D,M_{1}\right)=k_{2}\frac{D}{D+D_{50}}M_{1}$.

### Statistical inference

*In vivo* kinetic data of both virus and macrophage populations were extracted using WebPlotDigitizer (version 4.4) from [18]. Female BALB/c mice were intranasally infected with HP (A/1918 H1N1 and A/Thailand/16/2004 H5N1) and LP (A/Texas/36/91 H1N1 and A/Thailand/SP/83/2004 H5N1) influenza viruses, and lungs were harvested for viral load and macrophage measurement at various time points post-infection. Three mice were measured per time point for infection with each viral strain.

We applied a Bayesian inference method to fit the dynamic model (detailed in **Mathematical Model**) to the virological and macrophage data from [18]. The macrophages measured in the experiment included all three subtypes and therefore the sum of the subtypes, i.e., *M*_*R*_ + *M*_1_ + *M*_2_ in the model, were fitted to the macrophage data. The choice of which parameters were estimated by model fitting and which parameters were fixed was based on the experimental evidence presented in previous studies and the focus in this study on identifying mechanisms by which HP and LP viruses differ. In our study, 14 parameters were estimated (parameters: *β*_*HP*_, *β*_*LP*_, *q*_*FI*,*HP*_, *q*_*FI*,*LP*_, *q*_*FM*,*HP*_, *q*_*FM*,*LP*_, *s*_*V*,*HP*_, *s*_*V*,*LP*_, $q_{HP}^{\prime }$, $q_{LP}^{\prime }$, *κ*_*A*,*HP*_, *κ*_*A*,*LP*_, *V*_0_, *s*_*M*_). We chose to estimate the viral infectivity rates (i.e., *β*_*HP*_ and *β*_*LP*_) because Fukuyama et al. [11] have shown that a mutation in the cleavage site of the viral hemagglutinin (HA) protein could enhance both viral entry and viral infection efficiency in the host. Moreover, studies in [9, 11, 43, 44] have shown that viral expressed NS1 protein in high-pathogenic influenza strains can attenuate interferon response by reducing interferon production, based on which we chose to estimate the interferon production rates, *q*_*FI*,*HP*_, *q*_*FI*,*LP*_, *q*_*FM*,*HP*_ and *q*_*FM*,*LP*_ in the model, for HP and LP strains. Third, an extensive macrophage response, such as excessive numbers of macrophages, is one of the hallmarks associated with high-pathogenic viral infection [18]. Estimates of the macrophage recruitment rates, *s*_*V*,*HP*_ and *s*_*V*,*LP*_, allowed us to examine different macrophage dynamics in HP and LP infections. Fourth, there is evidence showing that macrophages have a reduced ability to engulf viruses in HP infection than in LP infection [17]. Therefore, we chose to estimate the engulfment rates of viruses by macrophages (i.e., $q_{HP}^{\prime }$ and $q_{LP}^{\prime }$) in HP and LP infections. Fifth, we chose to estimate the rates of neutralisation of HP and LP viruses by antibodies (i.e., *κ*_*A*,*HP*_ and *κ*_*A*,*LP*_) on HP and LP viruses as it was shown in [56] that antibody responses displayed a significant difference between HP and LP influenza viral infections. Sixth, the initial viral load (*V*_0_) was assumed (based on experimental procedures) to be the same for HP and LP and estimated. Finally, the recruitment rate of macrophages in the absence of viral infection (*s*_*M*_) was also assumed to be the same for HP and LP and estimated because the experimental data to which our model fitted were generated from inbred mice [18].

All other parameters (*k*_1_, *k*_2_, *k*_−1_, *k*_−2_, *δ*_*MR*_, *δ*_*MA*_, *D*_50_, *V*_50_, *α*, *g*_*T*_, *T*_0_, *δ*_*I*_, *δ*_*V*_, *κ*_*F*_, *κ*_*E*_, *κ*_*D*_, *δ*_*D*_, *p*_*I*_, *δ*_*F*_, *ϕ*, *ξ*_*R*_, *ℓ*) were fixed. This was done for a number of reasons. Firstly, there is no experimental evidence for a difference between HP and LP and for these parameters. Secondly, our focus is on estimating differences between HP and LP strains (by evaluating ratios of posterior estimates for parameters). Therefore, fixing other parameters at biologically plausible values (with sensitivity analyses to test for robustness of conclusions) helps to minimise computational and convergence issues in the Bayesian analysis pipeline typical of high-dimensional systems in which we may expect strong parameter correlations. Posterior estimates for model parameters must be considered in this light, that is, conditional on the choice of fixed parameters.

Parameter values for the fixed parameters were selected based on literature and/or previous estimation studies and were provided in S2 Text. Note that the fixed values of three parameters (*D*_50_, *V*_50_ and *α*) were based on a study of tuberculosis infection [52] and might differ in the context of influenza infection. To investigate the influence of the three fixed parameters on model-fitting results and conclusions, we conducted a sensitivity analysis. For example, we decreased (S12 Fig) or increased (S13 Fig) the parameter *D*_50_ from the default value by one order of magnitude and found that the parameter had no influence on the conclusions made based on the fitting results using the default value. Similar sensitivity analyses on other fixed parameters, e.g., decreasing *V*_50_ (S14 Fig) or increasing *V*_50_ (S15 Fig), and decreasing *α* (S16 Fig) or increasing *α* (S17 Fig), also confirmed that those fixed parameters had no influence on the conclusions.

The prior distributions for the estimated model parameters are given in S2 Text. The distribution of the observed log-transformed viral load and macrophage data is assumed to be a normal distribution with a mean value given by the model simulation results and standard deviation (SD) parameter with a prior distribution of a normal distribution with a mean of 0 and an SD of 1.

Model fitting was performed in R (version 4.0.2) and Stan (Rstan 2.21.0). Samples were drawn from the joint posterior distribution of the model parameters using Hamiltonian Monte Carlo (HMC) optimized by the No-U-Turn Sampler (NUTS) (see [25] for details). In particular, we used three chains with different starting points and ran 3000 iterations for each chain. The first 1000 iterations were discarded as burn-in, and we retained 6000 samples in total from the 3 chains (2000 for each). Detailed diagnostics and results can be found in S1 Text.

### Model prediction

Based on estimated posterior samples, we predict the maximal percentage of epithelial cell loss, the cumulative dead cells and the relative contribution of macrophages to viral clearance. The maximal percentage of epithelium loss is given by

$$\begin{array}{cc} & 1-min\left(T\left(t\right)+R\left(t\right)\right)/T_{0}\times 100\%, \end{array}$$ (11)

where *T*(*t*) and *R*(*t*) are the number of susceptible and refractory epithelial cells during infection, and *T*_0_ is the initial number of available susceptible cells and the carrying capacity of epithelial cells population. The area under the dead cell curve (*AUC*_*D*_) is given by

$$\begin{array}{cc} & AUC_{D}=\int_{0}^{\tau }D\left(t\right)dt, \end{array}$$ (12)

where *τ* is a cut-off day for calculation, and we set *τ* = 10 to cover viral and macrophage dynamics shown in [18]. *D*(*t*) is simulated time series of dead cells. *AUC*_*D*_ is defined to be the area under the time-series curve of the dead cells *D* over a period of time (Eq 12) and measures the cumulative number of dead cells that are left in the system at different times post-infection. We use the quantity to indicate the severity of infection. The relative contribution of macrophages to viral clearance is given by

$$\begin{array}{cc} & \frac{q^{\prime }M_{R}\left(t\right)V\left(t\right)}{\delta_{V}V\left(t\right)+q^{\prime }M_{R}\left(t\right)V\left(t\right)+\kappa_{A}t^{4}/\left(t^{4}+t_{A}^{4}\right)V\left(t\right)}, \end{array}$$ (13)

where *M*_*R*_(*t*) and *V*(*t*) are the number of resting macrophages and viral loads during infection. The prediction of tissue damage and the reproduction number were computed using 6000 posterior samples by solving the ordinary differential equations solver ode15s in MATLAB R2022a with a relative tolerance of 1 × 10^−5^ and an absolute tolerance of 1 × 10^−10^. The initial values were (*M*_*R*_, *M*_1_, *M*_2_, *T*, *L*, *I*, *V*, *F*, *R*, *D*) = (*s*_*M*_/*δ*_*MR*_, 0, 0, *T*_0_, 0, 0, *V*_0_, 0, 0, 0). All visualization was performed in R (version 4.0.2), and codes to produce all figures are available at https://github.com/keli5734/Rcode-pathogenicity.

### A simulation-estimation study

A simulation-estimation study was conducted to explore if the availability of time-series data on macrophage kinetics (in addition to viral kinetic data) for model fitting improves the estimates of model parameters. We generated two synthetic data sets by simulating from our model. The first data set included both viral kinetic and macrophage data while the second data set included only viral kinetic data. By applying our Bayesian statistical inference methods to these data sets we demonstrated that inclusion of macrophage data enabled accurate estimation of the recruitment rate of macrophages, and inference on the timing and strength of macrophage activation during influenza viral infection. In contrast, with only viral kinetic data available, these quantities were not able to be recovered reliably. Hence, the availability of viral load and macrophage data enhances our ability to understand macrophage-virus interactions and estimate the contribution of macrophages to viral clearance. Details on the simulation-estimation study can be found in S3 Text.

## Supporting information

S1 Fig Posterior distributions of parameters for H1N1 viruses. Green bars indicate the posterior density for the HP strain and purple bars indicate the posterior density for the LP strain. Green and purple dashed lines indicate the median estimation of each parameter for HP and LP, respectively. The prior distribution for each parameter is given by the black curve.
(PDF)

S2 Fig 2 Posterior distributions of parameters for H5N1 viruses. Green bars indicate the posterior density for the HP strain and purple bars indicate the posterior density for the LP strain. Green and purple dashed lines indicate the median estimation of each parameter for HP and LP, respectively. The prior distribution for each parameter is given by the black curve.
(PDF)

S3 Fig Prediction of tissue damage for H5N1 viruses. The violin plots (coloured) and boxplots (white) give the density and the median and extrema of the predicted quantity. (A) model prediction of the maximal epithelium loss for the HP (yellow) and green (LP) strains. (B) model prediction of the cumulative level of dead cells during the infection for both strains. ***p < 0.001. The calculation formula sees Eq (13) in the main text. All estimations are computed using 6000 posterior samples from model fitting.
(PDF)

S4 Fig Comparison of estimated model parameters between HP and LP strains of the H5N1 viruses. Histograms show the frequency of the ratios of estimated HP parameters over paired LP model parameters and are normalised to [0, 1]. The ratios are presented by distributions of 6000 samples because they are generated by 6000 posterior parameter values. The cumulative density functions (CDFs) are given by the solid lines, and the dashed lines indicate ratios = 0. All ratios are log10-scaled, such that ratios > 0 (dark green) suggest greater values of the HP parameters. Figs (A, B, C) show the ratios of viral infectivity, and interferon production rate from infected cells and activated macrophages, respectively. Figs (D, E, F) show the ratios of infection-induced macrophage recruitment rate, macrophage-mediated virus clearance rate and antibody neutralisation rate, respectively.
(PDF)

S5 Fig Correlations between estimated model parameters and tissue damage for the H5N1 viruses. Partial rank correlation coefficients (PRCC) are calculated with respect to (A) the ratio of max epithelium loss between HP and LP strains, and (B) the ratio of the cumulative dead cells between HP and LP strains of H5N1 viruses. The two red dashed lines represent the statistically insignificant values of PRCC. Calculations are based upon 6000 posterior samples from model fitting.
(PDF)

S6 Fig The relative contribution of macrophages to viral clearance in the HP and LP strains of the H5N1 viruses. The prediction interval (PI) is calculated based on the 6000 posterior samples from model fitting. The median trajectory is indicated by the black curve.
(PDF)

S7 Fig Parameter driving tissue damage for the LP H1N1 virus. Figs (A, B, C) give the sensitivity analyses of the impact of *β*, *q*_*FI*_ and *s*_*V*_ on maximal epithelium loss. Figs (D, E, F) show the impact of the same three model parameters on the cumulative dead cells.
(PDF)

S8 Fig The proportion of epithelium loss during HP and LP H1N1 viral infections. The calculation of epithelium loss is given in the main text. All estimations are computed using 6000 posterior samples from model fitting. The purple curve indicates the median trajectory.
(PDF)

S9 Fig The proportion of epithelium loss during HP and LP H5N1 viral infections. The calculation of epithelium loss is given in the main text. All estimations are computed using 6000 posterior samples from model fitting. The purple curve indicates the median trajectory.
(PDF)

S10 Fig Detailed macrophage dynamics during HP and LP H1N1 viral infections. Y-axis gives the proportion of each type of macrophage to the overall number of macrophages at each measuring time. Grey lines are macrophage trajectories calculated based on 6000 posterior samples from model fitting, and the median trajectory is indicated by the red curve.
(PDF)

S11 Fig Detailed macrophage dynamics during HP and LP H5N1 viral infections. Y-axis gives the proportion of each type of macrophage to the overall number of macrophages at each measuring time. Grey lines are macrophage trajectories calculated based on 6000 posterior samples from model fitting, and the median trajectory is indicated by the red curve.
(PDF)

S12 Fig Sensitivity analysis. *D*_50_ decreases an order of magnitude from the baseline value.
(PDF)

S13 Fig Sensitivity analysis. *D*_50_ increases an order of magnitude from the baseline value.
(PDF)

S14 Fig Sensitivity analysis. *V*_50_ decreases an order of magnitude from the baseline value.
(PDF)

S15 Fig Sensitivity analysis. *V*_50_ increases an order of magnitude from the baseline value.
(PDF)

S16 Fig Sensitivity analysis. *α* decreases an order of magnitude from the baseline value.
(PDF)

S17 Fig Sensitivity analysis. *α* increases an order of magnitude from the baseline value.
(PDF)

S1 Text Convergence diagnostics for the MCMC chains. (DOCX)

S2 Text Parameter tables. (DOCX)

S3 Text A simulation-estimation study. (DOCX)
