# Supplementary figures and images for: Enhanced viral infectivity and reduced interferon production are associated with high pathogenicity for influenza viruses
Source: PLoS Comput Biol. 2023 Feb 9;19(2):e1010886. doi: 10.1371/journal.pcbi.1010886 (PMC9946260; doi:10.1371/journal.pcbi.1010886)

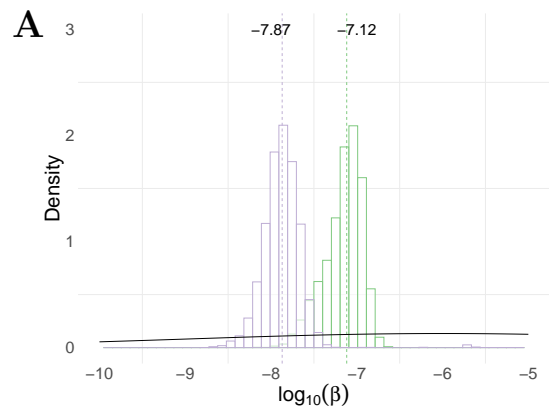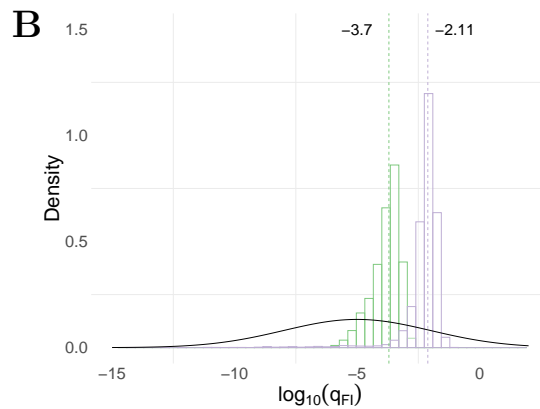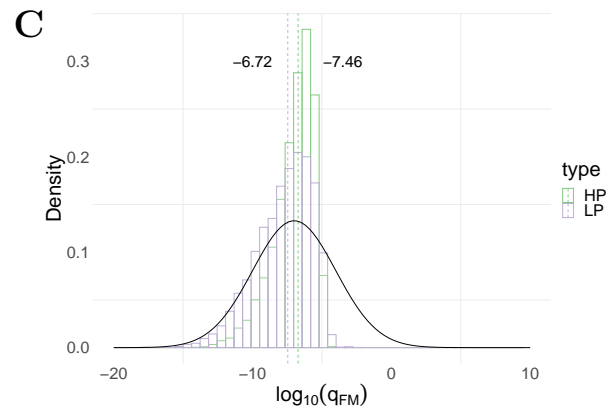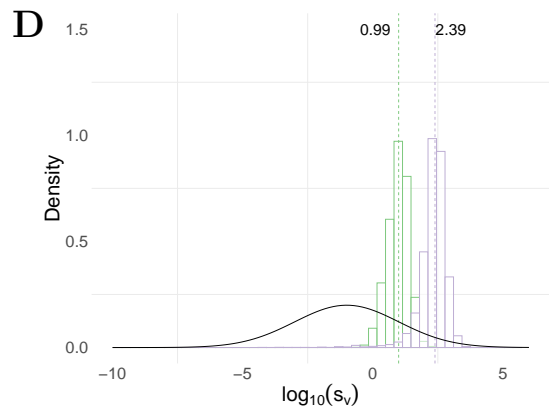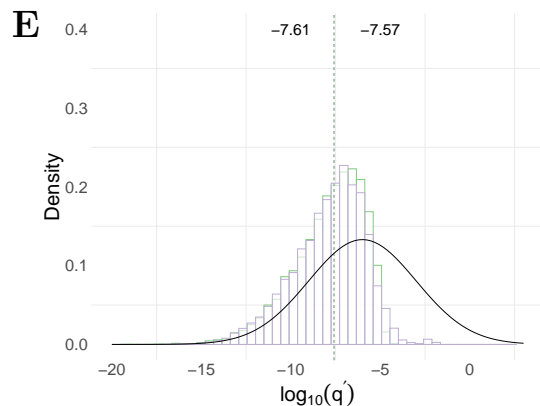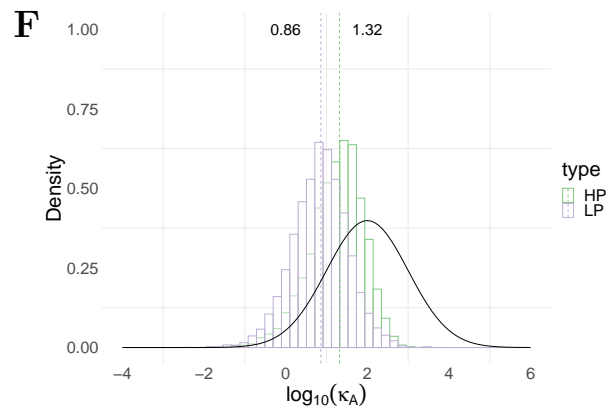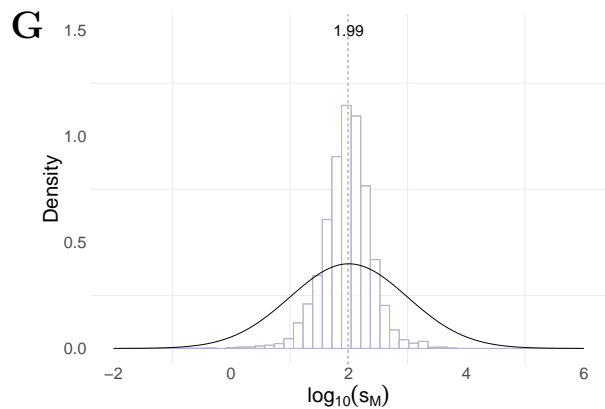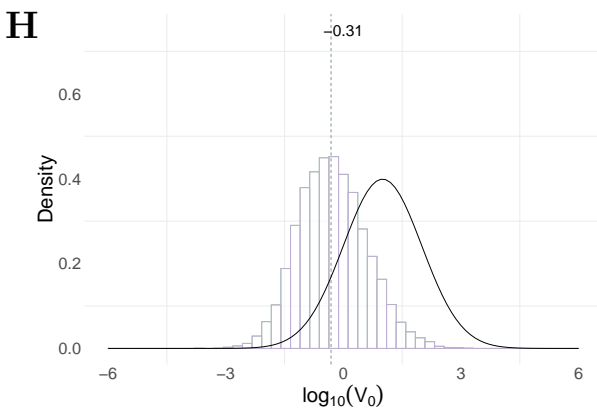

Supplement: S1 Fig — Green bars indicate the posterior density for the HP strain and purple bars indicate the posterior density for the LP strain. Green and purple dashed lines indicate the median estimation of each parameter for HP and LP, respectively. The prior distribution for each parameter is given by the black curve. (PDF) [file pcbi.1010886.s001.pdf]

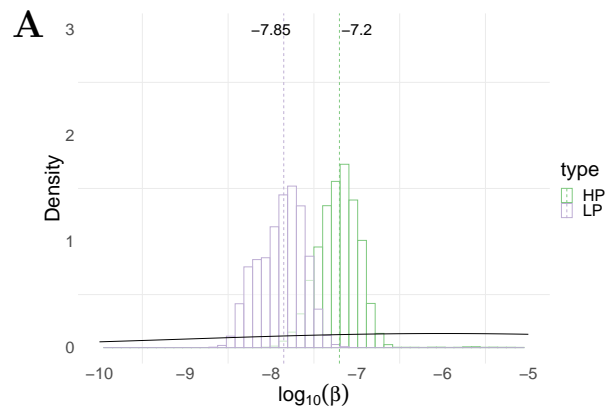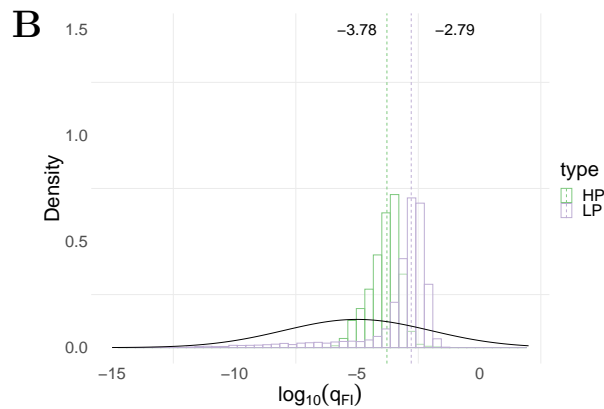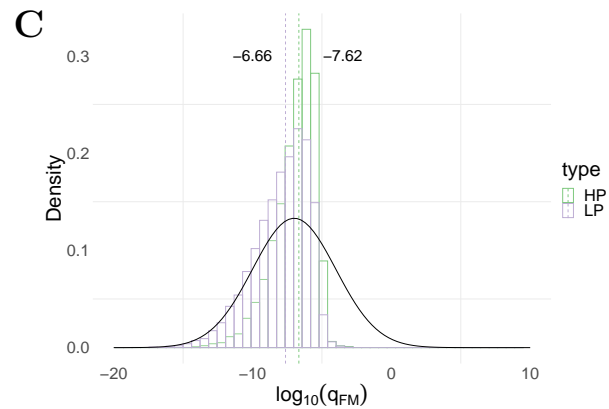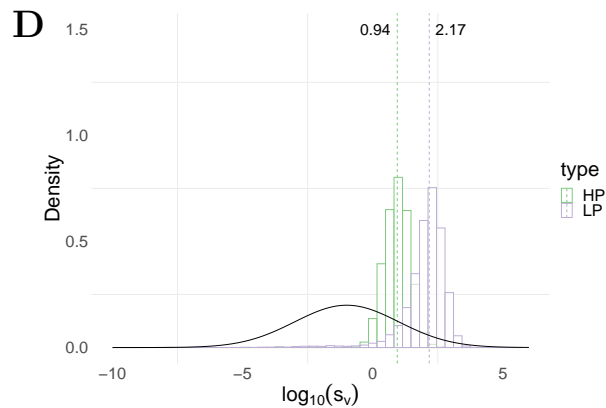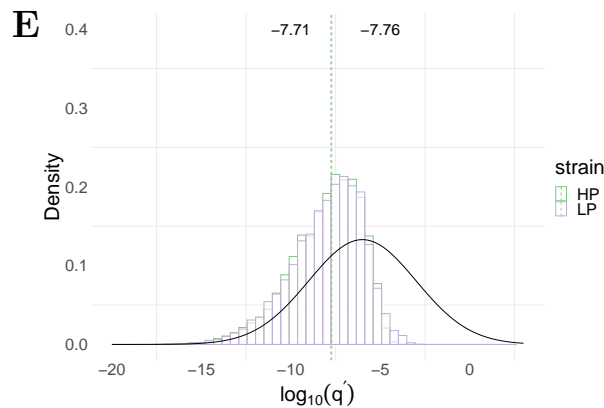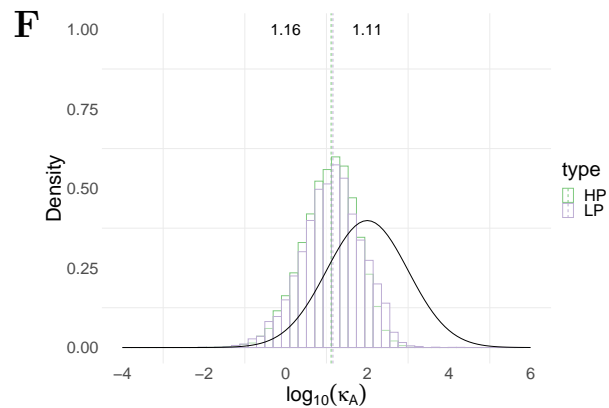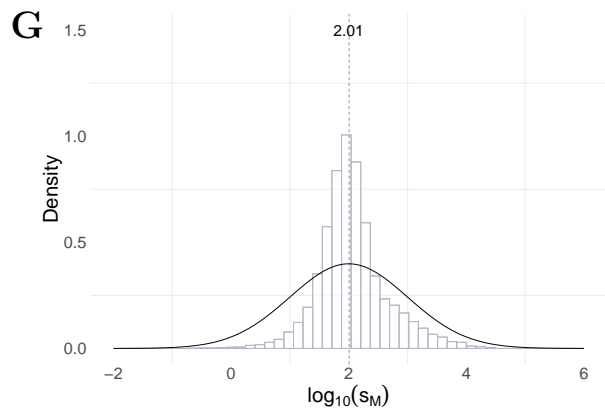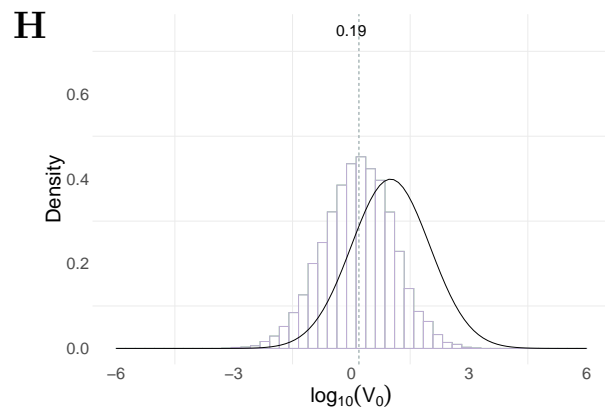

Supplement: S2 Fig — Green bars indicate the posterior density for the HP strain and purple bars indicate the posterior density for the LP strain. Green and purple dashed lines indicate the median estimation of each parameter for HP and LP, respectively. The prior distribution for each parameter is given by the black curve. (PDF) [file pcbi.1010886.s002.pdf]

**A****Max % of epithelium loss (H5N1)**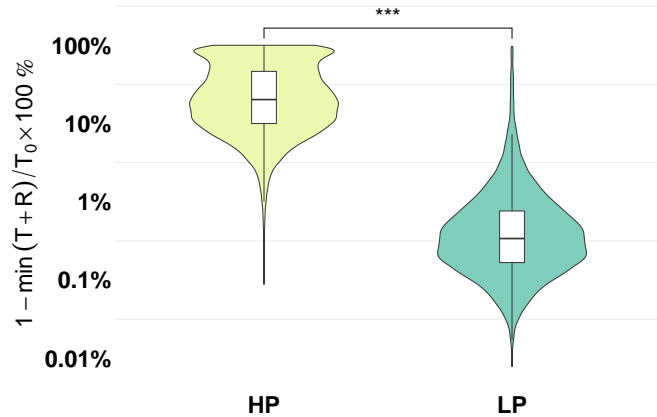**B****Cumulative level of dead cells (H5N1)**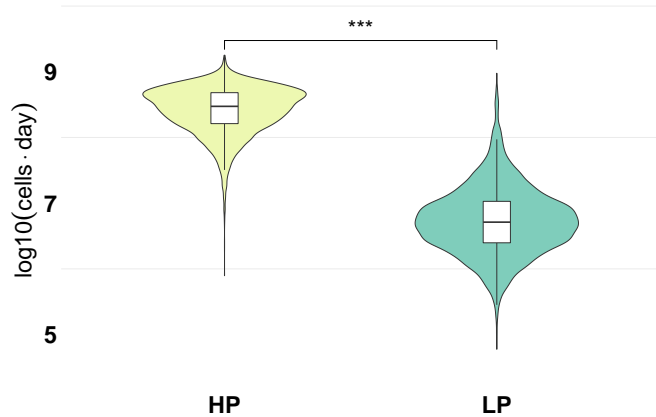

Supplement: S3 Fig — The violin plots (coloured) and boxplots (white) give the density and the median and extrema of the predicted quantity. (A) model prediction of the maximal epithelium loss for the HP (yellow) and green (LP) strains. (B) model prediction of the cumulative level of dead cells during the infection for both strains. ***p < 0.001. The calculation formula sees Eq (13) in the main text. All estimations are computed using 6000 posterior samples from model fitting. (PDF) [file pcbi.1010886.s003.pdf]

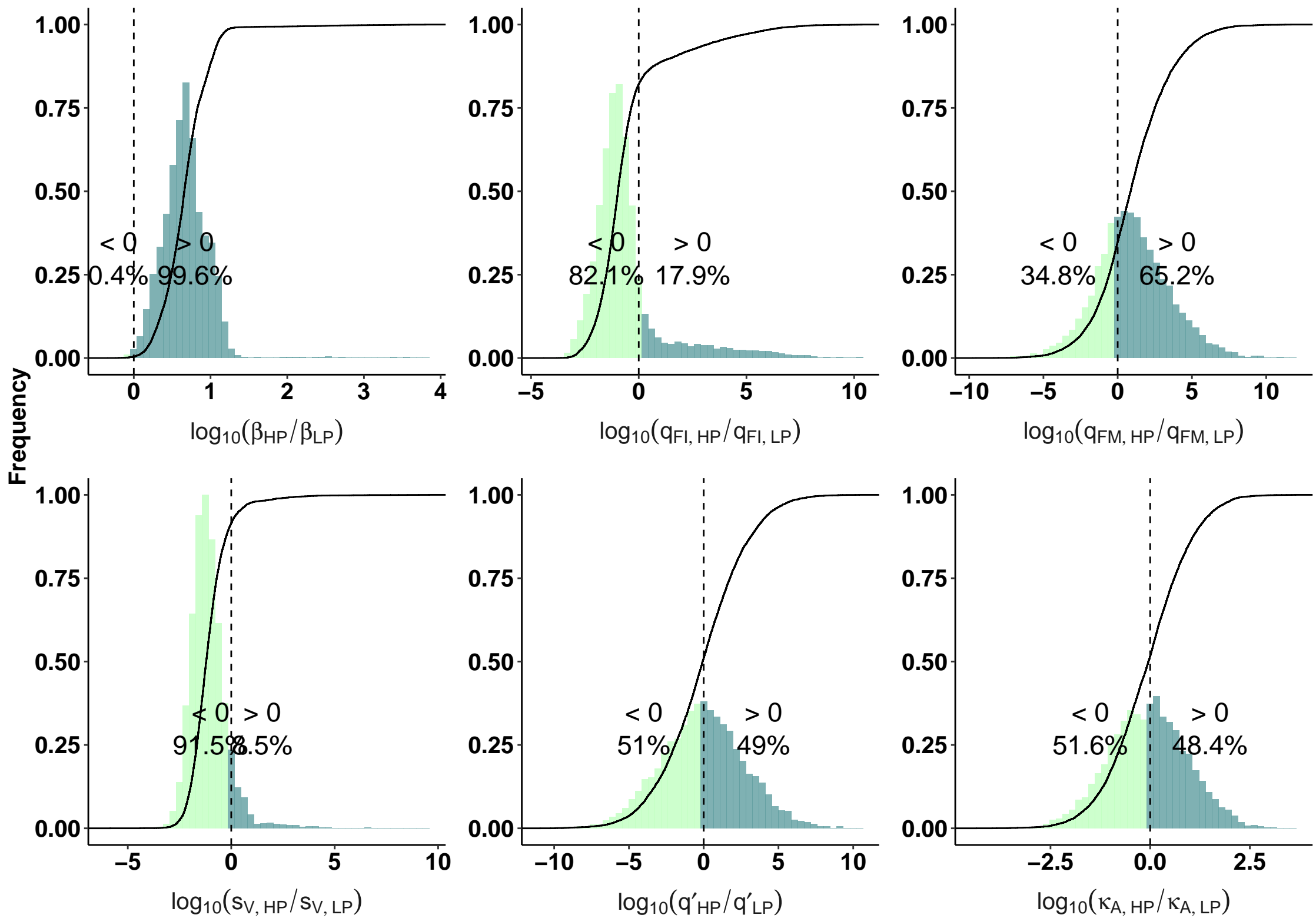

Supplement: S4 Fig — Histograms show the frequency of the ratios of estimated HP parameters over paired LP model parameters and are normalised to [0, 1]. The ratios are presented by distributions of 6000 samples because they are generated by 6000 posterior parameter values. The cumulative density functions (CDFs) are given by the solid lines, and the dashed lines indicate ratios = 0. All ratios are log10-scaled, such that ratios > 0 (dark green) suggest greater values of the HP parameters. Figs (A, B, C) show the ratios of viral infectivity, and interferon production rate from infected cells and activated macrophages, respectively. Figs (D, E, F) show the ratios of infection-induced macrophage recruitment rate, macrophage-mediated virus clearance rate and antibody neutralisation rate, respectively. (PDF) [file pcbi.1010886.s004.pdf]

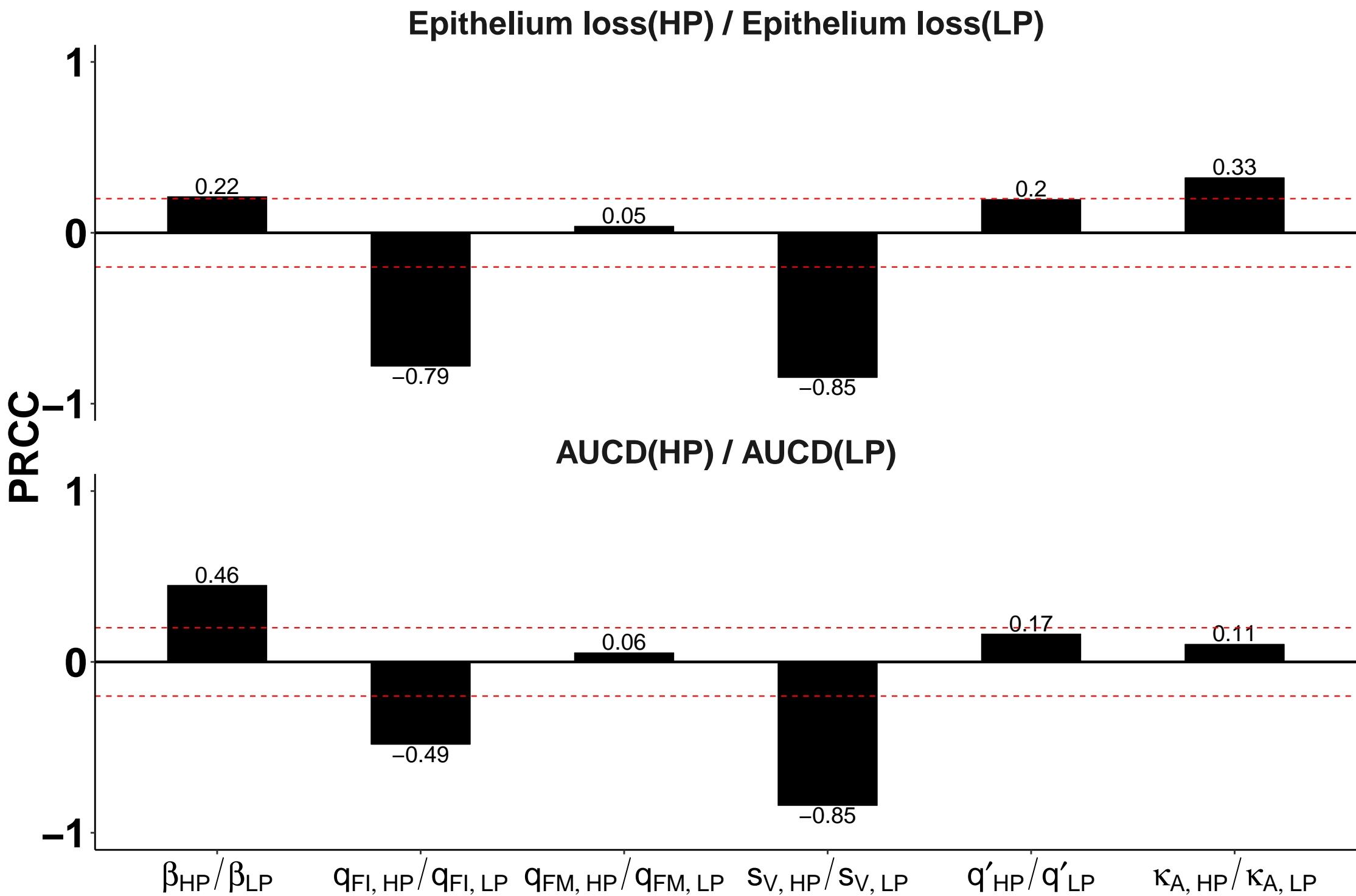

Supplement: S5 Fig — Partial rank correlation coefficients (PRCC) are calculated with respect to (A) the ratio of max epithelium loss between HP and LP strains, and (B) the ratio of the cumulative dead cells between HP and LP strains of H5N1 viruses. The two red dashed lines represent the statistically insignificant values of PRCC. Calculations are based upon 6000 posterior samples from model fitting. (PDF) [file pcbi.1010886.s005.pdf]

# Macrophage contribution to viral clearance (H5N1)

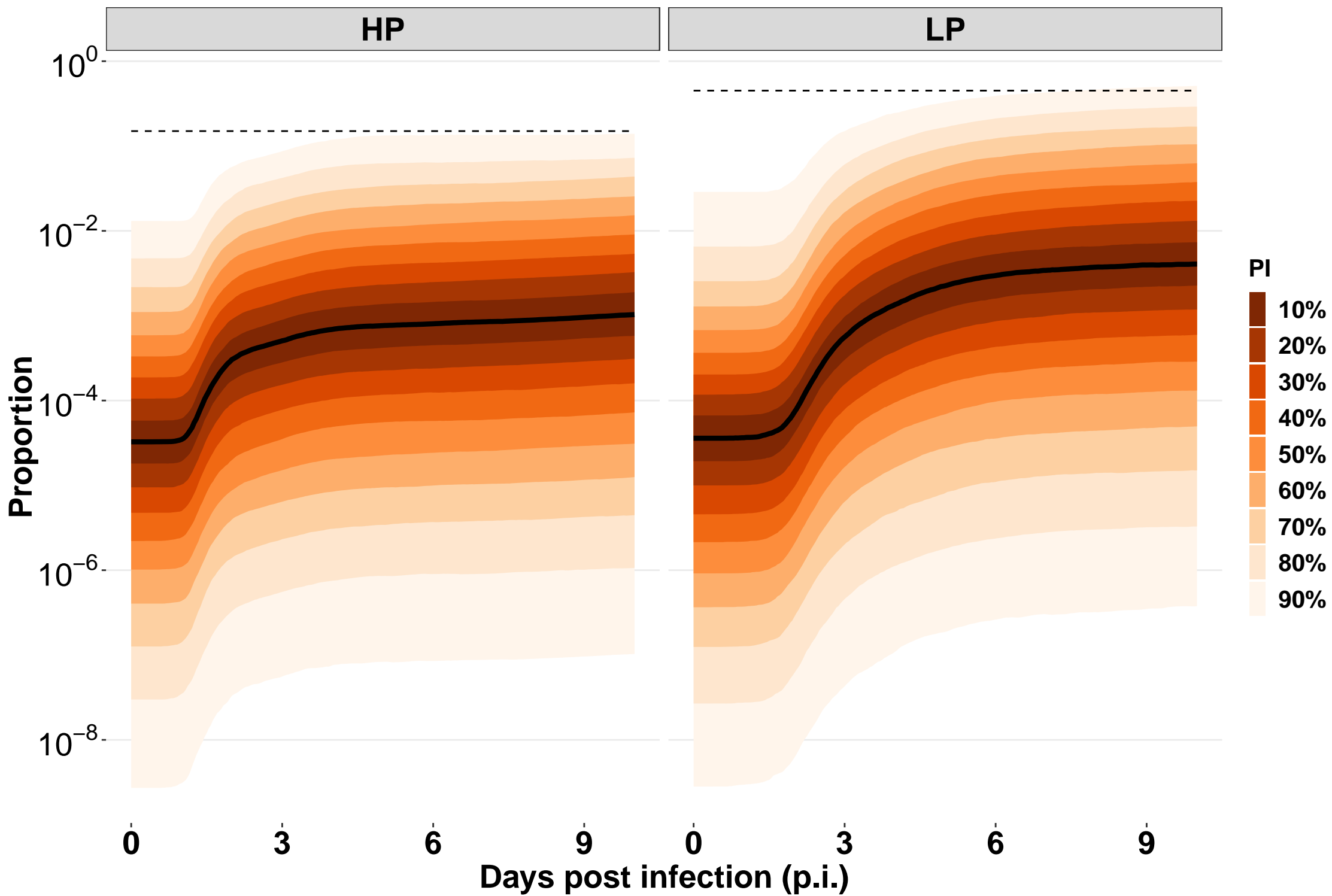

Supplement: S6 Fig — The prediction interval (PI) is calculated based on the 6000 posterior samples from model fitting. The median trajectory is indicated by the black curve. (PDF) [file pcbi.1010886.s006.pdf]

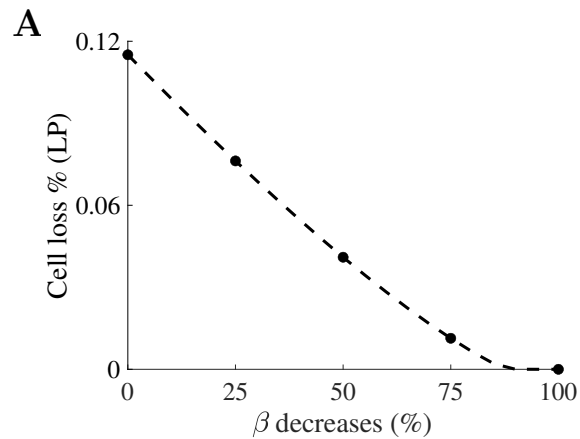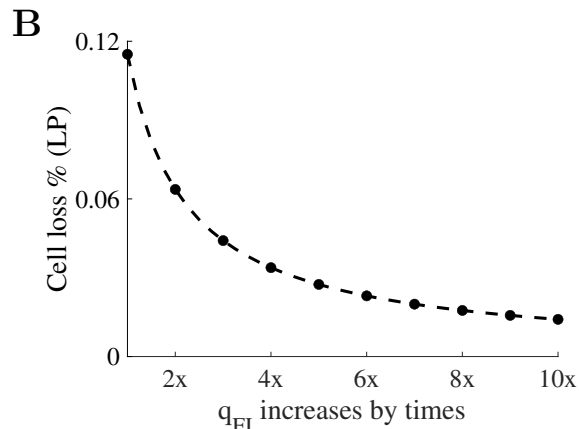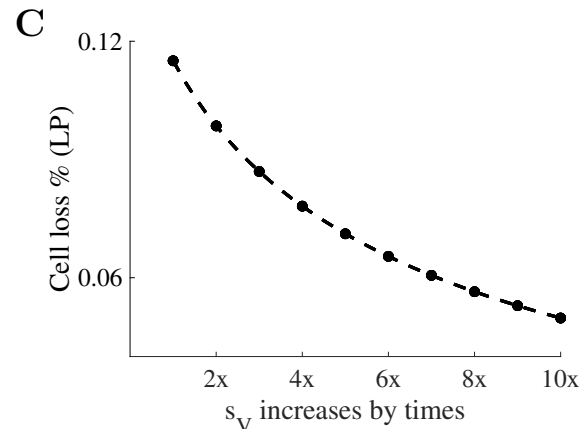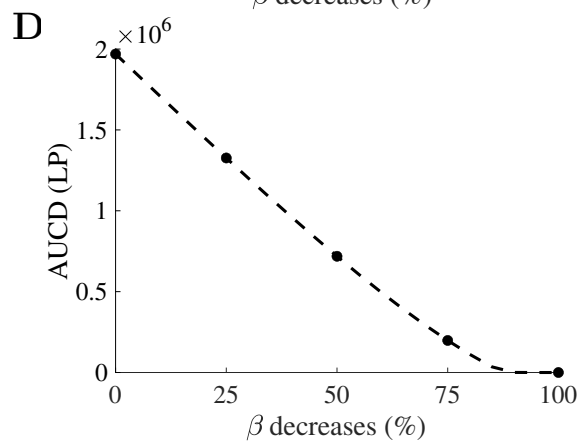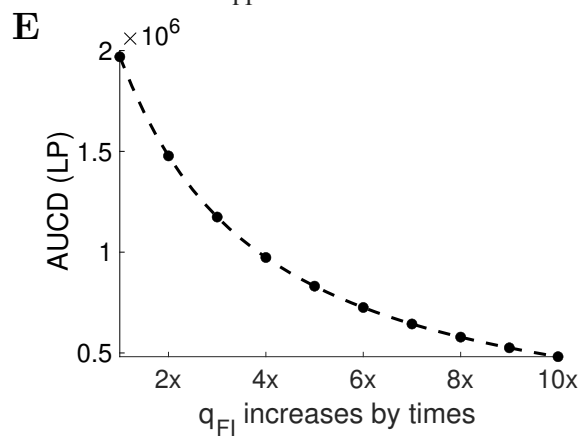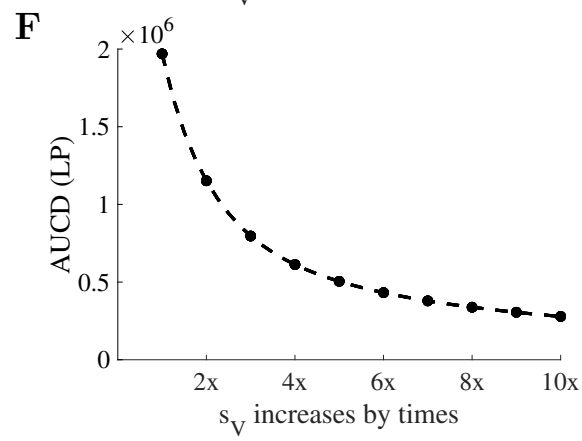

Supplement: S7 Fig — Figs (A, B, C) give the sensitivity analyses of the impact of β, qFI and sV on maximal epithelium loss. Figs (D, E, F) show the impact of the same three model parameters on the cumulative dead cells. (PDF) [file pcbi.1010886.s007.pdf]

# Epithelium loss during infection (H1N1)

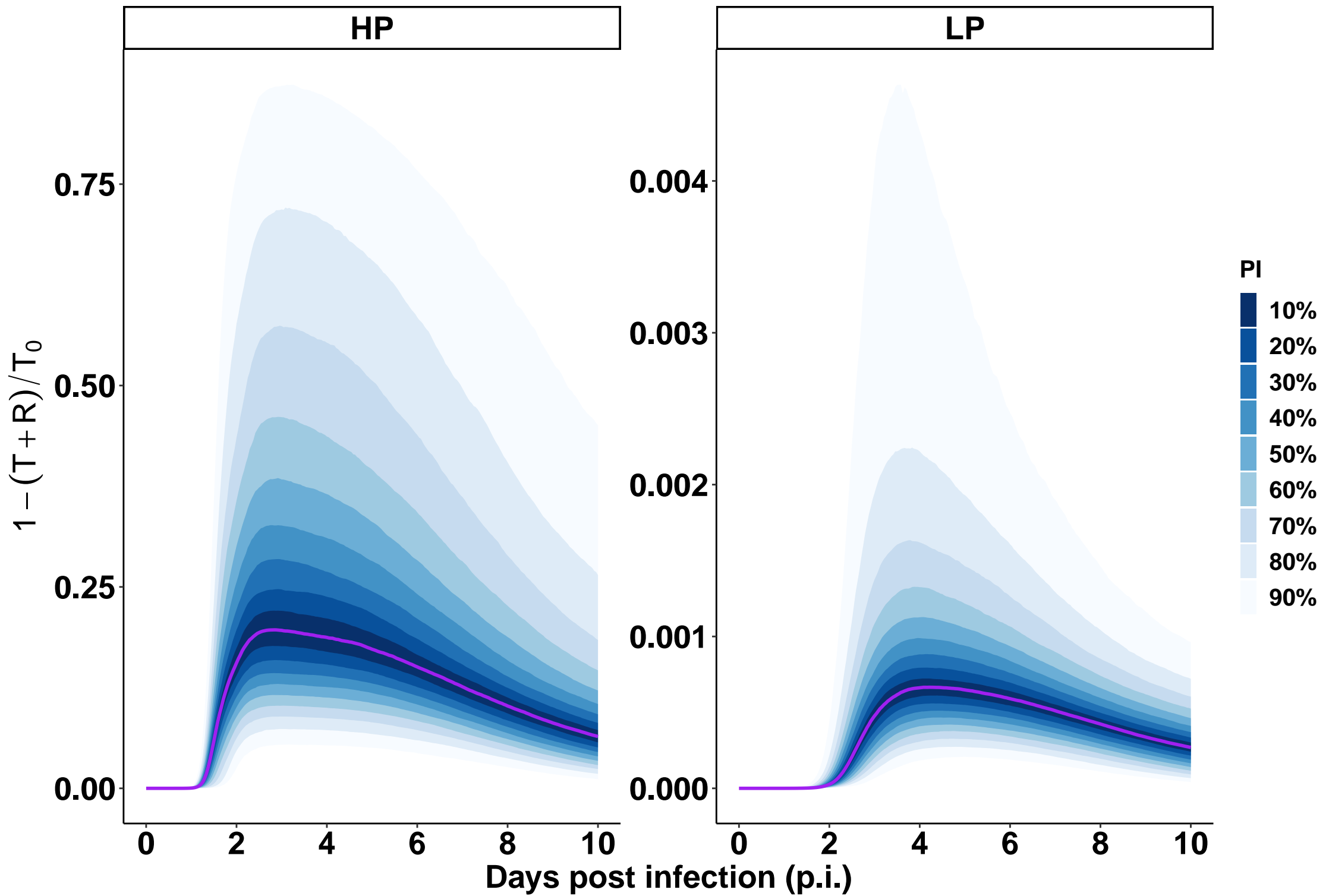

Supplement: S8 Fig — The calculation of epithelium loss is given in the main text. All estimations are computed using 6000 posterior samples from model fitting. The purple curve indicates the median trajectory. (PDF) [file pcbi.1010886.s008.pdf]

# Epithelium loss during infection (H5N1)

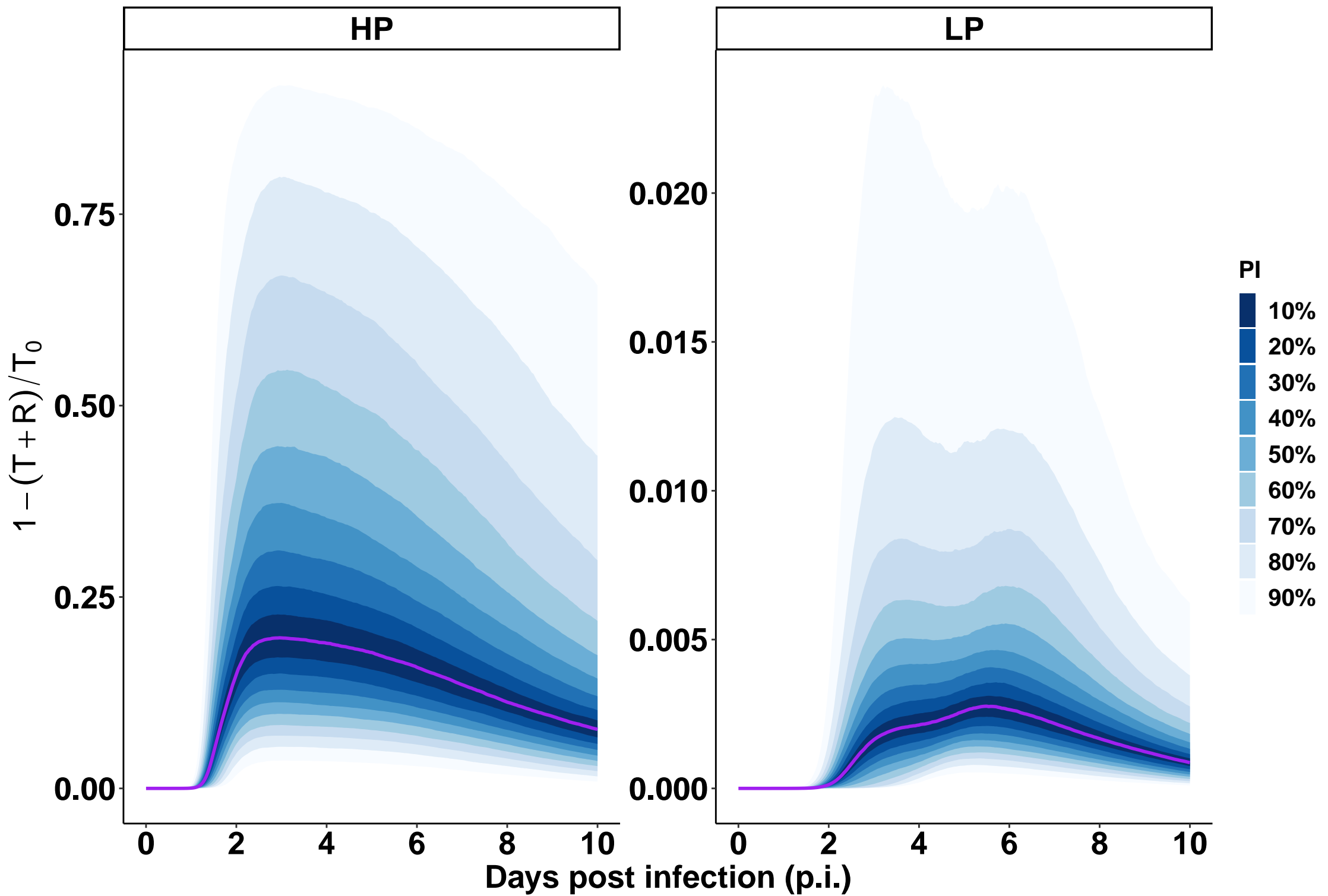

Supplement: S9 Fig — The calculation of epithelium loss is given in the main text. All estimations are computed using 6000 posterior samples from model fitting. The purple curve indicates the median trajectory. (PDF) [file pcbi.1010886.s009.pdf]

Detailed macrophage dynamics (H1N1)

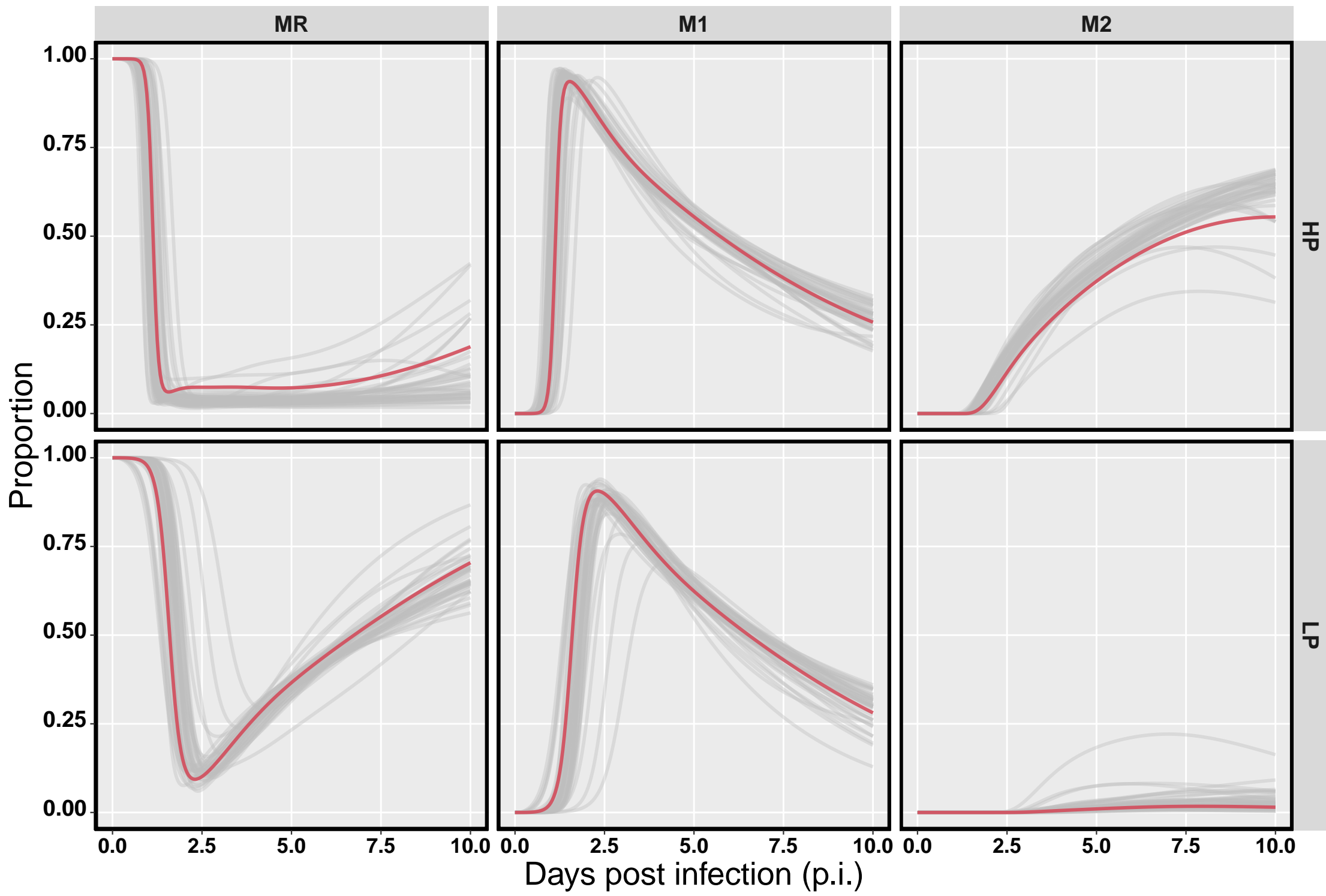

Supplement: S10 Fig — Y-axis gives the proportion of each type of macrophage to the overall number of macrophages at each measuring time. Grey lines are macrophage trajectories calculated based on 6000 posterior samples from model fitting, and the median trajectory is indicated by the red curve. (PDF) [file pcbi.1010886.s010.pdf]

Detailed macrophage dynamics (H5N1)

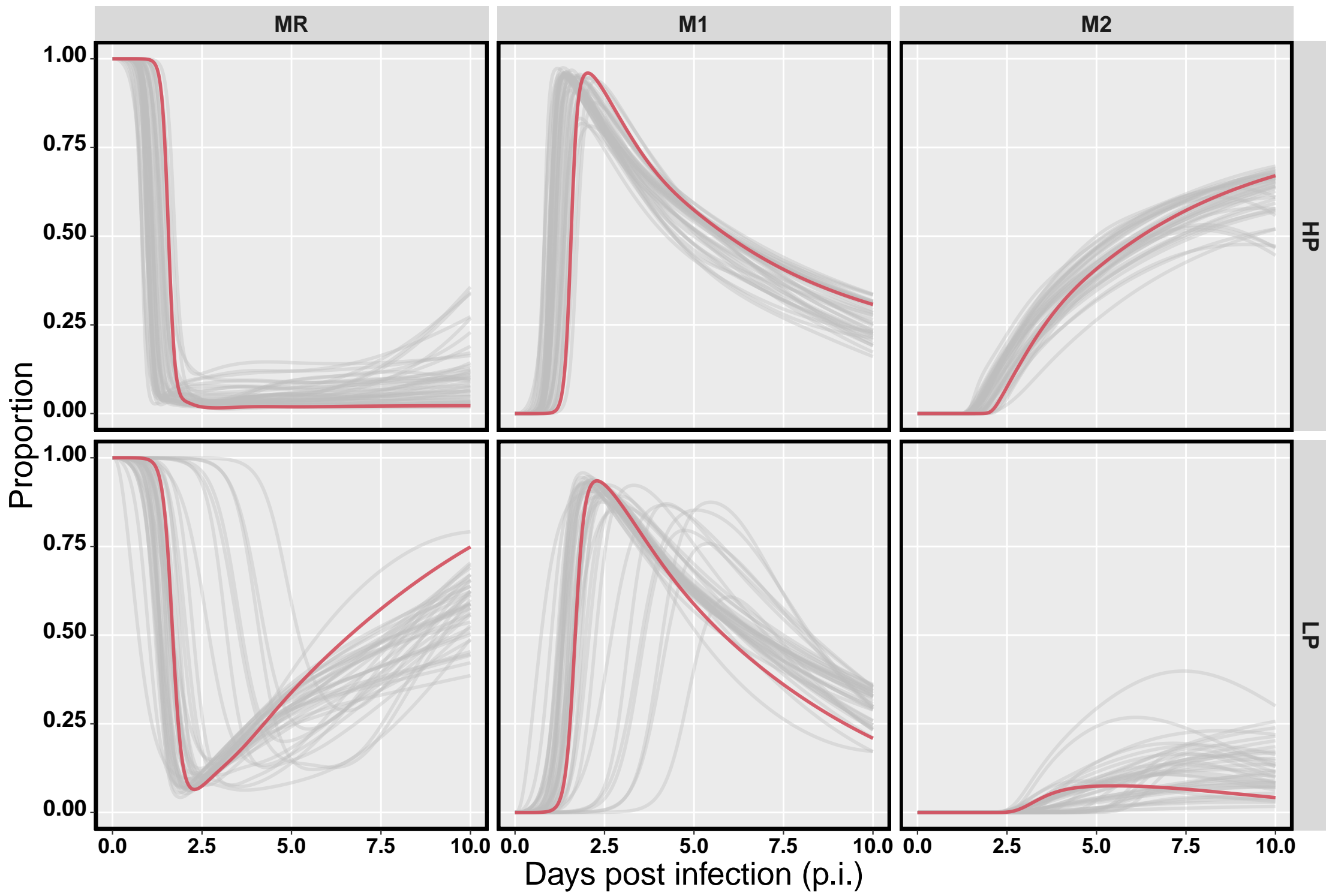

Supplement: S11 Fig — Y-axis gives the proportion of each type of macrophage to the overall number of macrophages at each measuring time. Grey lines are macrophage trajectories calculated based on 6000 posterior samples from model fitting, and the median trajectory is indicated by the red curve. (PDF) [file pcbi.1010886.s011.pdf]

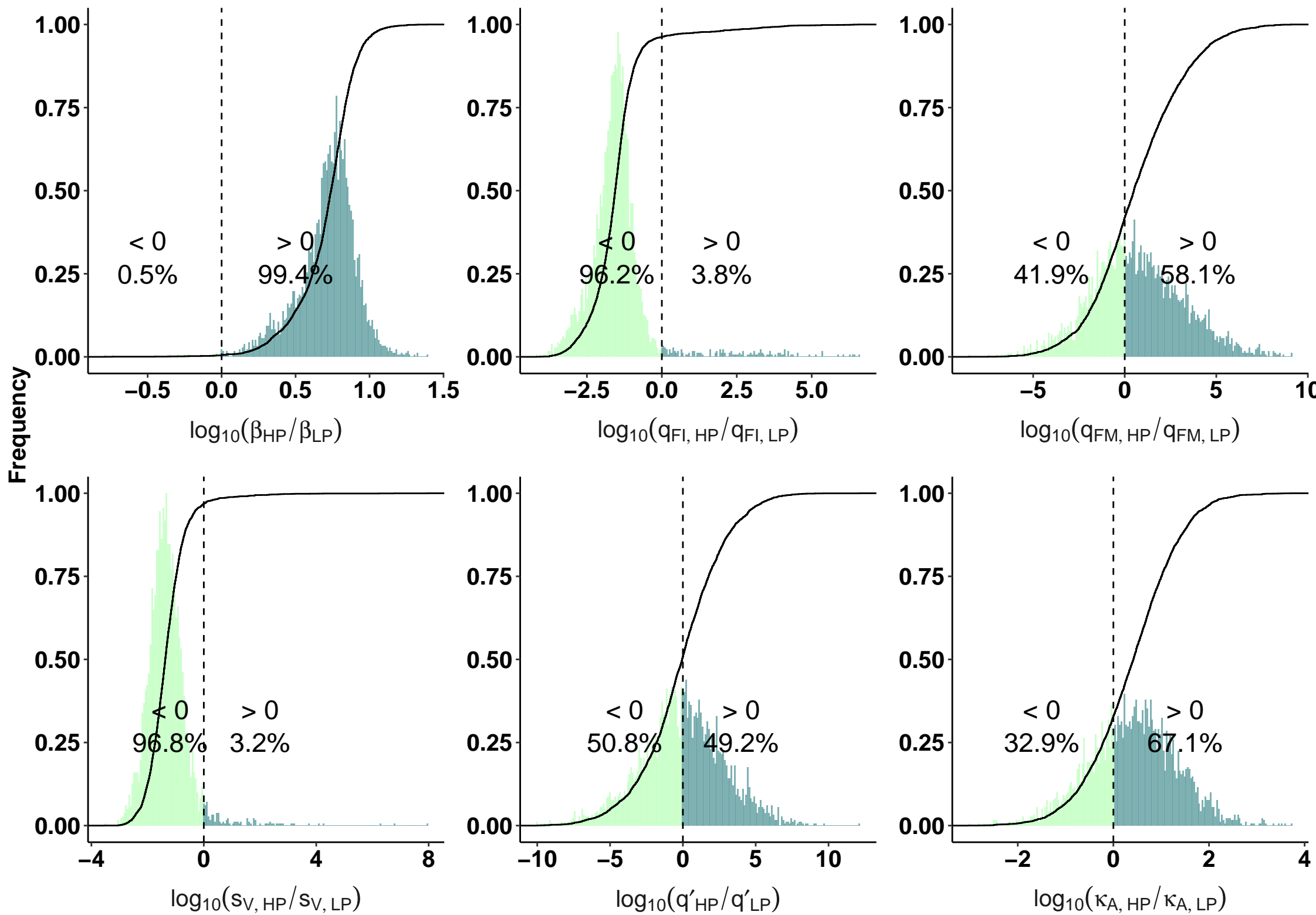

Supplement: S12 Fig — D50 decreases an order of magnitude from the baseline value. (PDF) [file pcbi.1010886.s012.pdf]

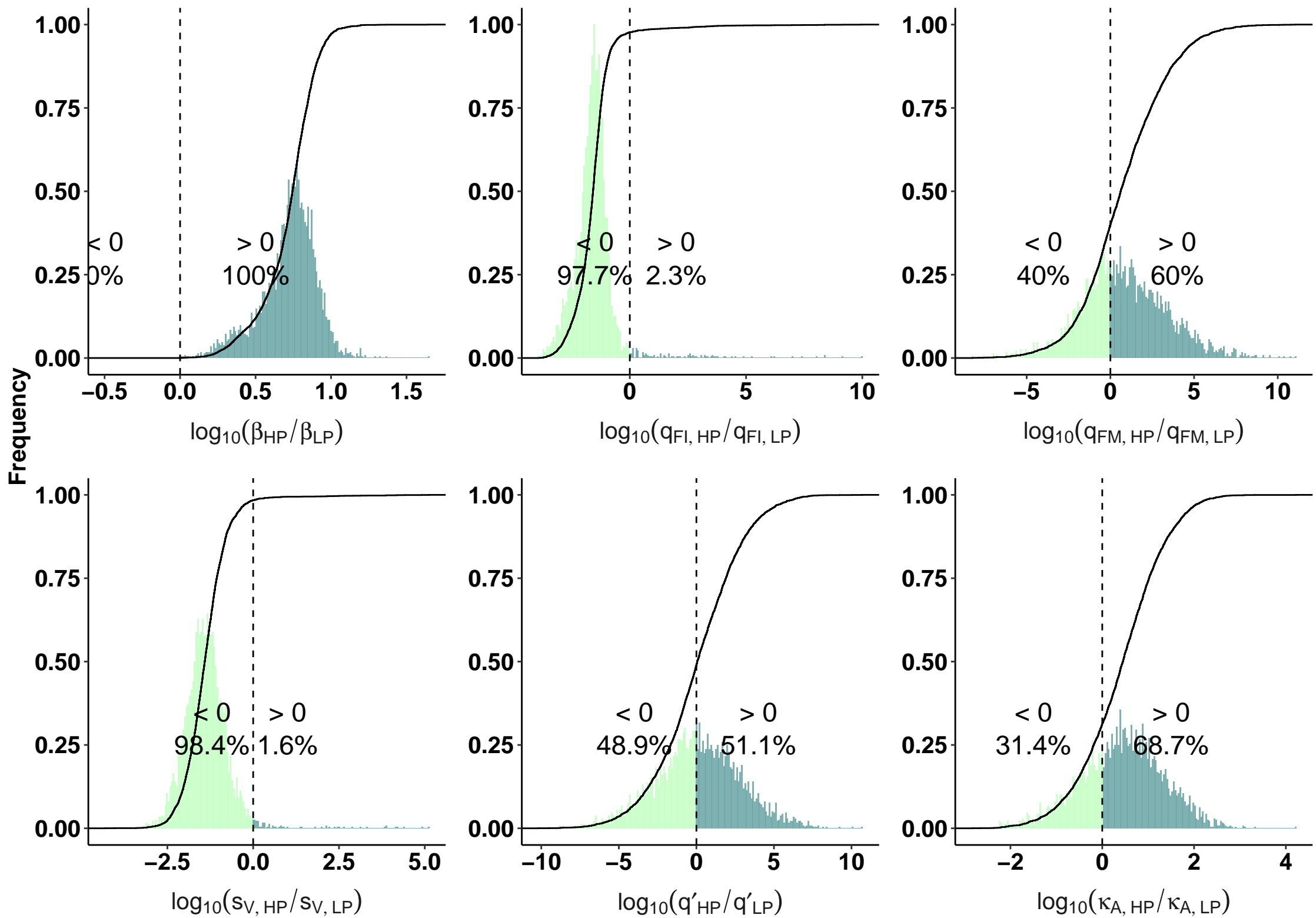

Supplement: S13 Fig — D50 increases an order of magnitude from the baseline value. (PDF) [file pcbi.1010886.s013.pdf]

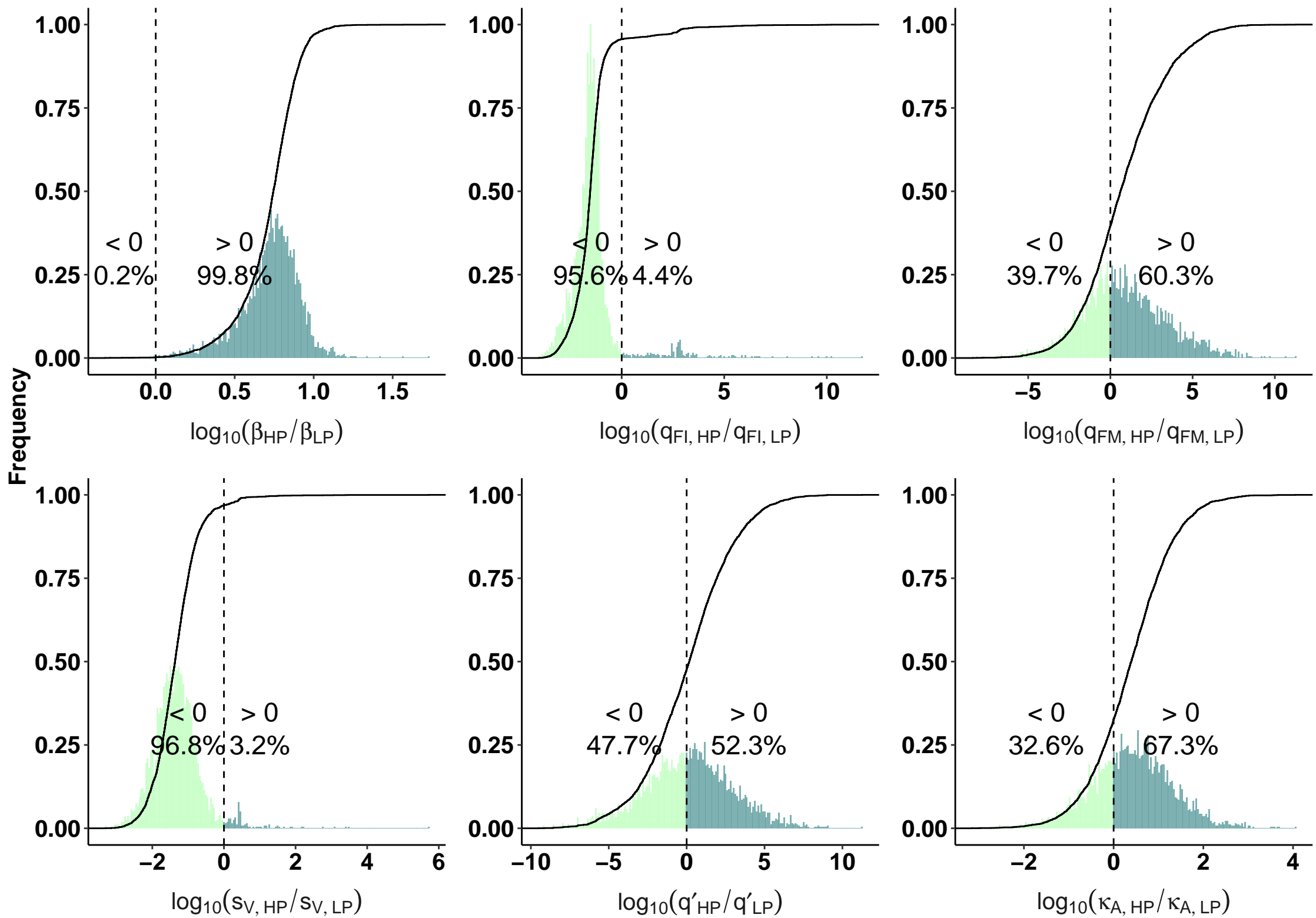

Supplement: S14 Fig — V50 decreases an order of magnitude from the baseline value. (PDF) [file pcbi.1010886.s014.pdf]

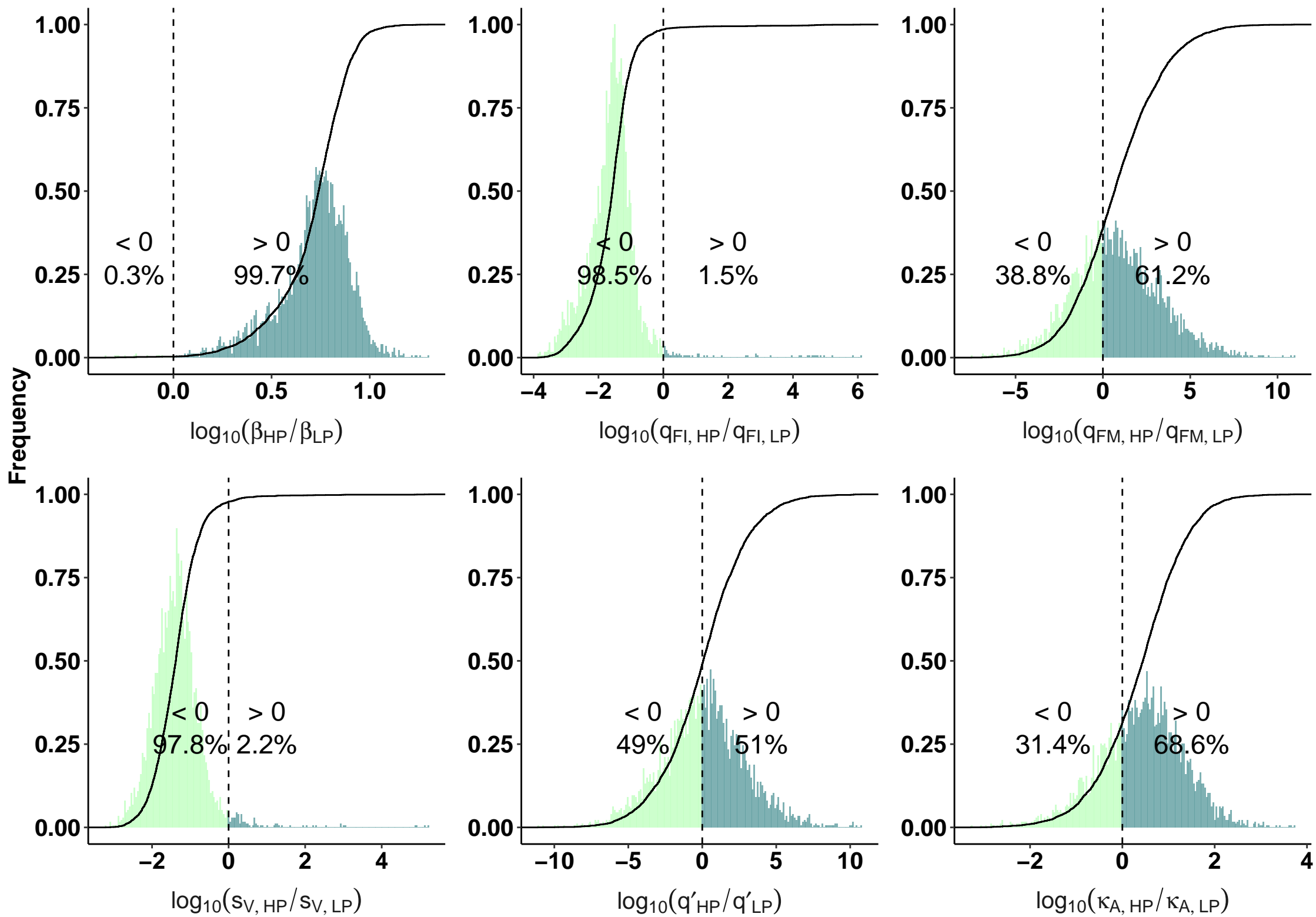

Supplement: S15 Fig — V50 increases an order of magnitude from the baseline value. (PDF) [file pcbi.1010886.s015.pdf]

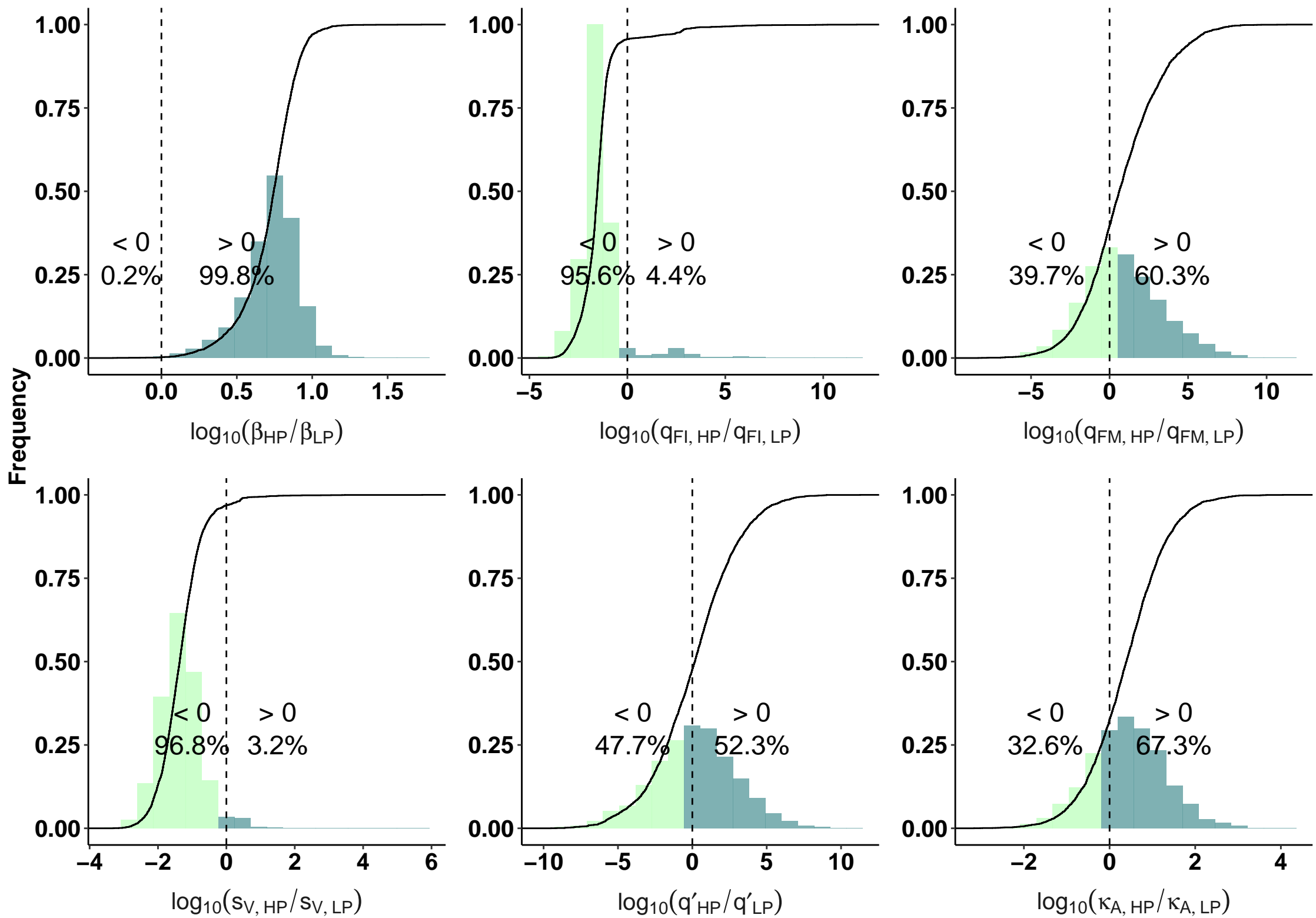

Supplement: S16 Fig — α decreases an order of magnitude from the baseline value. (PDF) [file pcbi.1010886.s016.pdf]

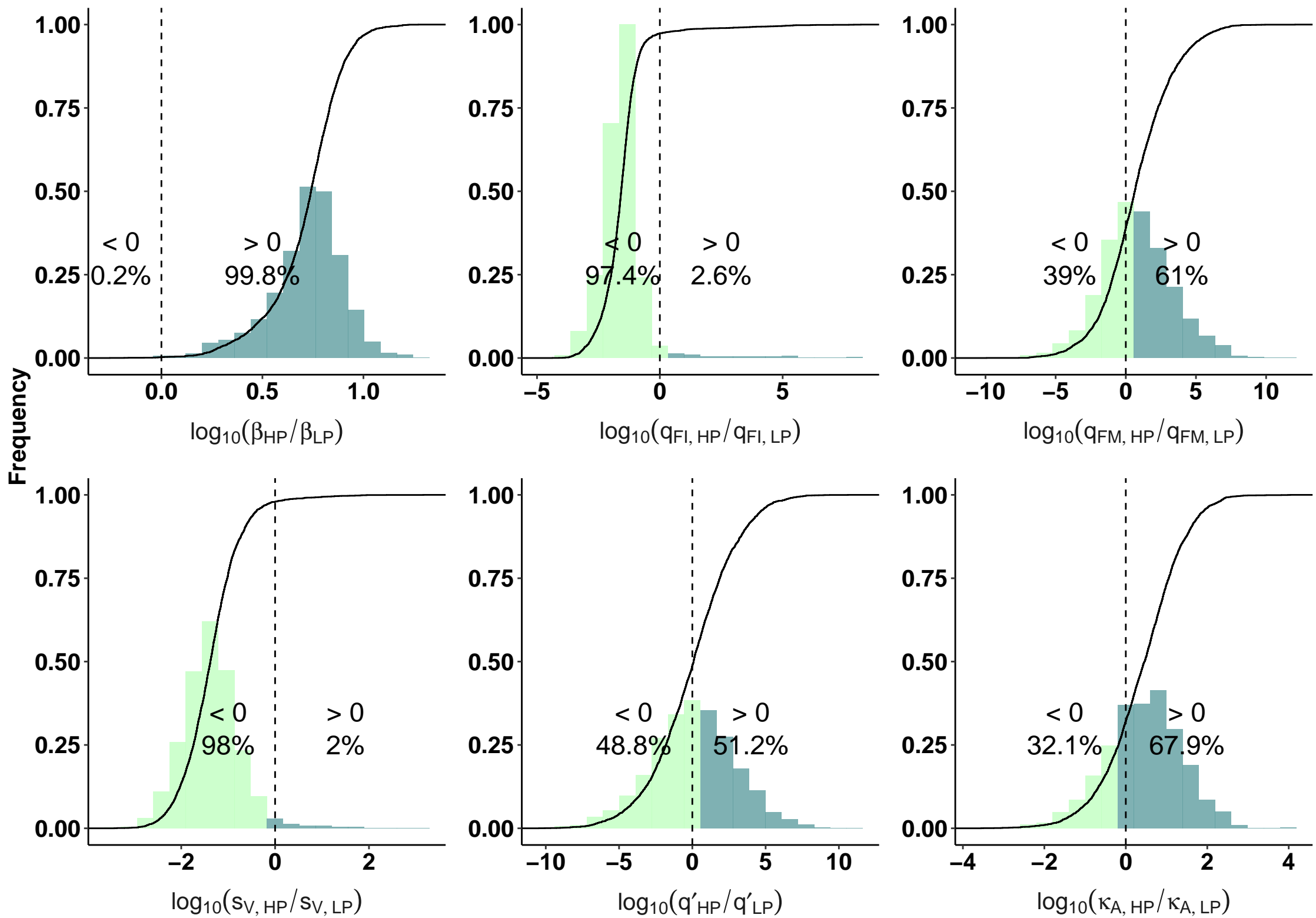

Supplement: S17 Fig — α increases an order of magnitude from the baseline value. (PDF) [file pcbi.1010886.s017.pdf]
