# Supplementary material for: Enhanced viral infectivity and reduced interferon production are associated with high pathogenicity for influenza viruses
Source: PLoS Comput Biol. 2023 Feb 9;19(2):e1010886. doi: 10.1371/journal.pcbi.1010886 (PMC9946260; doi:10.1371/journal.pcbi.1010886)
Supplement: S1 Text — (DOCX) [file pcbi.1010886.s018.docx]

**Enhanced infectivity and attenuation of interferon production are associated with high pathogenicity for influenza viruses**

Ke Li, James M McCaw, Pengxing Cao

S1 Text

Convergence diagnostics for the MCMC chains

Figures A and B show the trace plots for the evolution of estimated parameter vector over the iterations of 3 Markov chains for implementing H1N1 and H5N1 virus, respectively. For each chain, the iteration number is 4000 with the first 1000 samples as burn-in. We observe that all three chains do overlap together, indicating convergence has occurred. Tables A and B show the credible intervals, effective sample size and $\hat{r}$ of each estimated parameters for HP and LP strains of H1N1 or H5N1 virus, respectively. We find that the effective sample size is sufficient and  $\hat{r}$ is below 1.1 for every parameter, suggesting convergence.

**Figure A Trace plots of estimated parameters for the fitting of H1N1 viral and macrophage data.** Three chains were used with 4000 iterations, and the first 1000 iterations are burn-ins (grey area). All parameters are log-transformed. The parameter vector for HP $\Phi_{HP}=({log}_{10}( s_{V}), {log}_{10}\left( \beta\right), {log}_{10}\left( q_{FI} \right), {log}_{10}\left( q_{FM} \right), {log}_{10}\left( s_{M} \right), {log}_{10}\left( \kappa_{A} \right), {log}_{10}\left( q^{'} \right), {log}_{10}\left( V_{0} \right))$, and $\Phi_{LP}=\left( {log}_{10}\left( s_{V} \right), {log}_{10}\left( \beta\right), {log}_{10}\left( q_{FI} \right), {{log}_{10}(q}_{FM} \right), {log}_{10}\left( \kappa_{A} \right), {log}_{10}(q^{'}))$ for LP. We assume $s_{M}$ and $V_{0}$ are the same for both HP and LP strains. $\sigma_{1}$ and $\sigma_{2}$ are error structures for the prior distribution of standard deviation of the observed log-transformed viral load and macrophage data.

**Figure B Trace plots of estimated parameters for the fitting of H5N1 viral and macrophage data.**


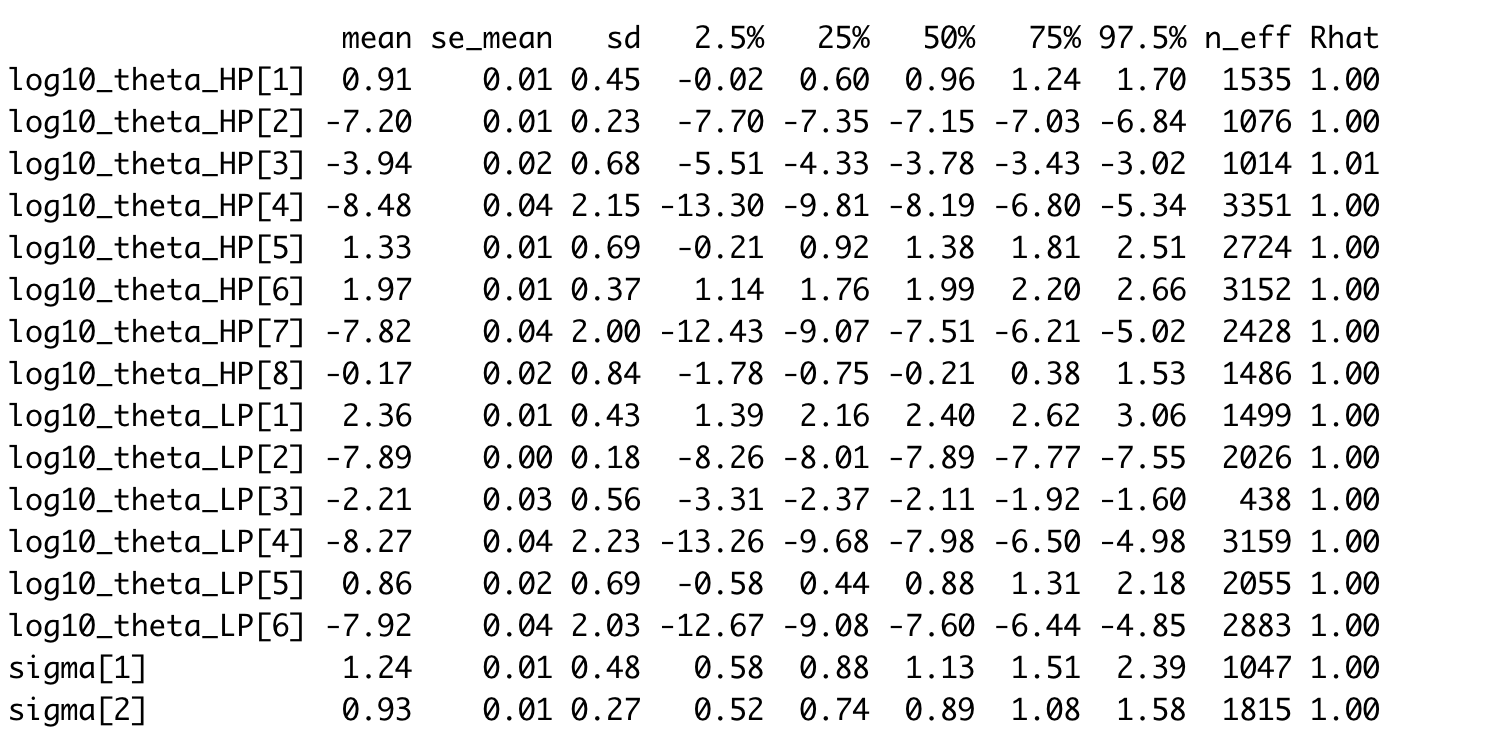


**Table A Credible intervals, effective sample sizes and**$\hat{\boldsymbol{r}}$ **for each estimated parameter of HP and LP strains for H1N1 viruses.** The first 1000 iterations are discarded as burn-in, leaving 6000 samples across the three chains. $\Phi_{HP}=({log}_{10}( s_{V}),{log}_{10}\left( \beta\right), {log}_{10}\left( q_{FI} \right),{log}_{10}\left( q_{FM} \right), {log}_{10}\left( s_{M} \right),{log}_{10}\left( \kappa_{A} \right), {log}_{10}\left( q^{'} \right),{log}_{10}\left( V_{0} \right))$, and $\Phi_{LP}=({log}_{10}(s_{V}),{log}_{10}( \beta),{log}_{10}(q_{FI}),{{log}_{10}(q}_{FM}),{log}_{10}(\kappa_{A}), {log}_{10}(q^{'}))$ for LP. $\sigma_{1}$ and $\sigma_{2}$ are error structures for the prior distribution of standard deviation of the observed log-transformed viral load and macrophage data.


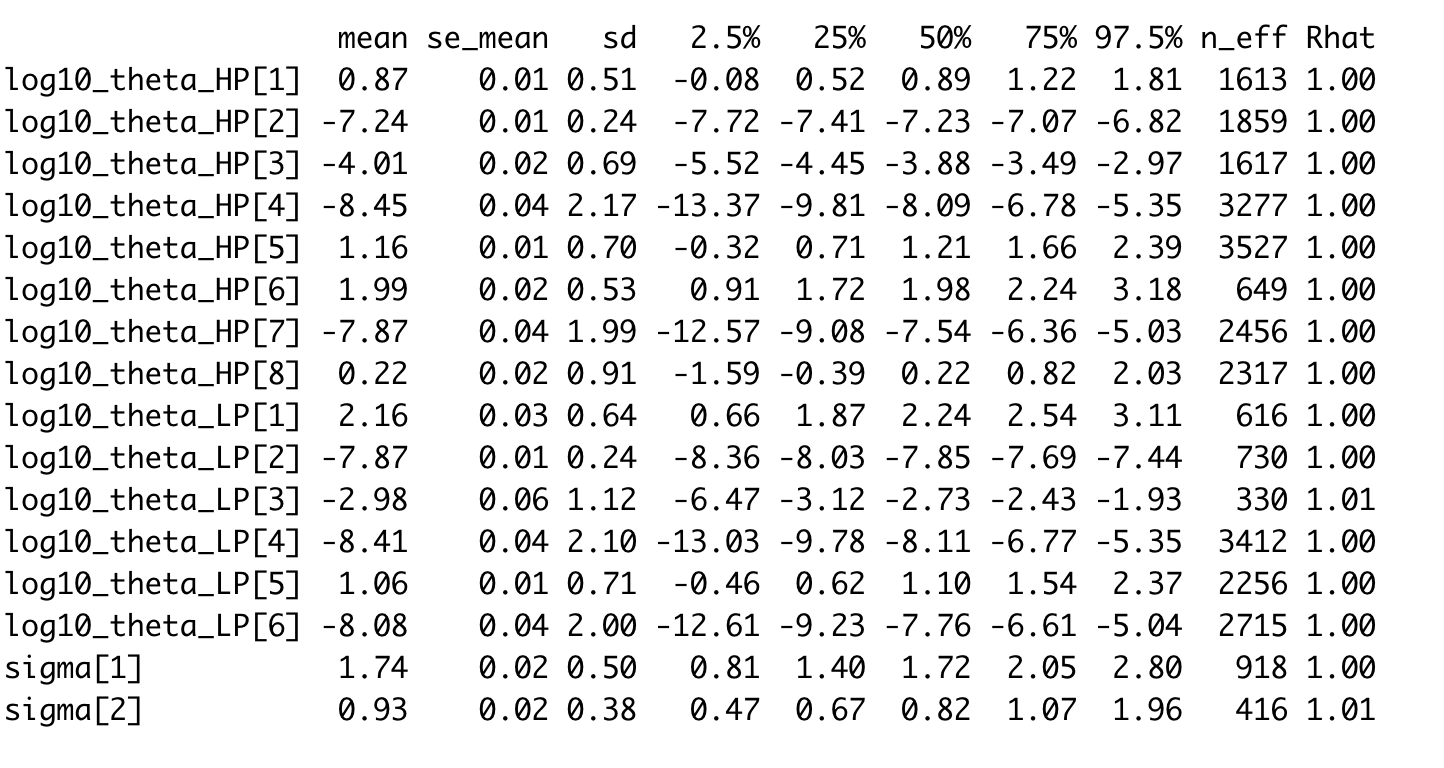


**Table B Credible intervals, effective sample sizes and**$\hat{\boldsymbol{r}}$ **for each estimated parameter of HP and LP strains for H5N1 viruses.**
