# Supplementary material for: Enhanced viral infectivity and reduced interferon production are associated with high pathogenicity for influenza viruses
Source: PLoS Comput Biol. 2023 Feb 9;19(2):e1010886. doi: 10.1371/journal.pcbi.1010886 (PMC9946260; doi:10.1371/journal.pcbi.1010886)
Supplement: S2 Text — (DOCX) [file pcbi.1010886.s019.docx]

**Enhanced infectivity and attenuation of interferon production are associated with high pathogenicity for influenza viruses**

Ke Li, James M McCaw, Pengxing Cao

S2 Text

Parameter tables

**Values for fixed Parameter table (Table S1)**

| Parameter | Description | Value [refs] | Unit |
| --- | --- | --- | --- |
| $\delta_{MR}$ | Decay rate of $M_{R}$macrophages | 1.1e-2 [9] | /day |
| $k_{-1}$ | Conversion rate from $M1$ to $M_{R}$ | 0.3 [9] | /day |
| $k_{-2}$ | Conversion rate from $M2$ to $M_{R}$ | 0.3 [9] | /day |
| $\delta_{MA}$ | Decay rate of activated $M1$and $M2$ macrophages | 1.1e-2 [9] | /day |
| $k_{1}$ | Conversion rate from $M_{R}$ to $M1$ | 0.4 [9] | /day |
| $k_{2}$ | Conversion rate from $M_{R}$ to $M2$ | 4e-5 [9] | /day |
| $V_{50}$ | Half saturation of viral load to activate $M1$ macrophages. | 1e+7 [9] | PFU/ml |
| $\alpha$ | Effectiveness of $M2$ attenuates $M_{R}$ to $M1$ | 1e-4 [9] | /cell |
| $D_{50}$ | Half saturation of dead cells | 1e+6 [9] | cell |
| $g_{T}$ | Regrowth rate of epithelium | 0.8 [6] | /day |
| $T_{max}$ | The maximal epithelium cells | 7e+7 [7] | cell |
| $\delta_{I}$ | Decay rate of infected cells | 2 [1,8,10] | /day |
| $\delta_{V}$ | Decay rate of virus | 5 [1,8,10] | /day |
| $\kappa_{F}$ | Clearance rate of infected cells by interferons | 3 [7] | /(day [$\mu_{F}$])  *[$\mu_{F}$] is the unit for interferon |
| $\kappa_{E}$ | Clearance rate of infected cells by CD8+T cells | 8 [7] | /day |
| $\kappa_{D}$ | Clearance rate of dead cells by $M1$ macrophages | 8e-7[9] | /(day cell) |
| $\delta_{D}$ | Decay rate of dead cells | 2 [12] | /day |
| $p_{I}$ | Viral production rate | 210 [7] | pfu/(ml cell day) |
| $\delta_{F}$ | Decay rate of interferons | 2 [7] | /day |
| $\phi$ | Conversion rate from $T$ to $R$ | 0.33 [7] | /(day [$\mu_{F}$]) |
| $\xi_{R}$ | Conversion rate from $R$ to $T$ | 2.6 [7] | /day |
| $t_{E}$ | Half saturation term of CD8+ T cell response | 5 | day |
| $t_{A}$ | Half saturation term of antibody response | 5 | day |
| $\mathcal{l}$ | Eclipse phase | 4 [5] | /day |

**Priors for estimated Parameter (Table S2)**

| Parameter | Description | Prior [refs] | Unit |
| --- | --- | --- | --- |
| ${log}_{10} {(s}_{V})$ | Recruitment rate of $M1$ due to infection | Normal(-1,2) [2,4,5,6,9] | /day |
| ${log}_{10} (\beta)$ | Viral infectivity rate | Normal(-6,3) [1,3,5,7,8,10,11] | /(pfu/ml day) |
| ${log}_{10} {(q}_{FI})$ | IFN production rate by infected cells | Normal(-6,3) [7,8] | [$\mu_{F}$]/(day cell) |
| ${log}_{10} (q_{FM})$ | IFN production rate by $M_{1}$ macrophages | Normal(-6,3) [12] | [$\mu_{F}$]/(day cell) |
| ${log}_{10} {(s}_{M})$ | Recruitment rate of macrophages in homeostasis | Normal(2,1) [2,4,5,6,9] | cell/day |
| ${log}_{10} (\kappa_{A})$ | Neutralization rate of antibodies on viruses | Normal(2,1) [7,8,10] | /day |
| ${log}_{10} (q')$ | Engulfment rate of macrophages on viruses | Normal(-6,3) | /(day cell) |
| ${log}_{10} \left( V_{0} \right)$ | Viral inoculation size | Normal(1,1) [13] | pfu/ml |

**Reference**

1. Baccam, P., Beauchemin, C., Macken, C.A., Hayden, F.G. and Perelson, A.S., 2006. Kinetics of influenza A virus infection in humans. *Journal of virology*, *80*(15), pp.7590-7599.
2. Eftimie, R. and Hamam, H., 2017. Modelling and investigation of the CD4+ T cells–macrophages paradox in melanoma immunotherapies. *Journal of theoretical biology*, *420*, pp.82-104.
3. Pawelek, K.A., Huynh, G.T., Quinlivan, M., Cullinane, A., Rong, L. and Perelson, A.S., 2012. Modeling within-host dynamics of influenza virus infection including immune responses. *PLoS computational biology*, *8*(6), p.e1002588.
4. Smith, A.M., McCullers, J.A. and Adler, F.R., 2011. Mathematical model of a three-stage innate immune response to a pneumococcal lung infection. *Journal of theoretical biology*, *276*(1), pp.106-116.
5. Smith, A.M., Adler, F.R., McAuley, J.L., Gutenkunst, R.N., Ribeiro, R.M., McCullers, J.A. and Perelson, A.S., 2011. Effect of 1918 PB1-F2 expression on influenza A virus infection kinetics. *PLoS computational biology*, *7*(2), p.e1001081.
6. Li, X., Jolly, M.K., George, J.T., Pienta, K.J. and Levine, H., 2019. Computational modeling of the crosstalk between macrophage polarization and tumor cell plasticity in the tumor microenvironment. *Frontiers in oncology*, *9*, p.10.
7. Cao, P., Wang, Z., Yan, A.W., McVernon, J., Xu, J., Heffernan, J.M., Kedzierska, K. and McCaw, J.M., 2016. On the role of CD8+ T cells in determining recovery time from influenza virus infection. *Frontiers in immunology*, *7*, p.611.
8. Yan, A.W., Zaloumis, S.G., Simpson, J.A. and McCaw, J.M., 2019. Sequential infection experiments for quantifying innate and adaptive immunity during influenza infection. *PLoS computational biology*, *15*(1), p.e1006568.
9. Wigginton, J.E. and Kirschner, D., 2001. A model to predict cell-mediated immune regulatory mechanisms during human infection with Mycobacterium tuberculosis. *The Journal of Immunology*, *166*(3), pp.1951-1967.
10. Miao, H., Hollenbaugh, J.A., Zand, M.S., Holden-Wiltse, J., Mosmann, T.R., Perelson, A.S., Wu, H. and Topham, D.J., 2010. Quantifying the early immune response and adaptive immune response kinetics in mice infected with influenza A virus. *Journal of virology*, *84*(13), pp.6687-6698.
11. Petrie, S.M., Guarnaccia, T., Laurie, K.L., Hurt, A.C., McVernon, J. and McCaw, J.M., 2013. Reducing uncertainty in within-host parameter estimates of influenza infection by measuring both infectious and total viral load. *PLoS One*, *8*(5), p.e64098.
12. Jenner, A.L., Aogo, R.A., Alfonso, S., Crowe, V., Deng, X., Smith, A.P., Morel, P.A., Davis, C.L., Smith, A.M. and Craig, M., 2021. COVID-19 virtual patient cohort suggests immune mechanisms driving disease outcomes. *PLoS pathogens*, *17*(7), p.e1009753.
13. Perrone, L.A., Plowden, J.K., García-Sastre, A., Katz, J.M. and Tumpey, T.M., 2008. H5N1 and 1918 pandemic influenza virus infection results in early and excessive infiltration of macrophages and neutrophils in the lungs of mice. *PLoS pathogens*, *4*(8), p.e1000115.
