# Supplementary material for: Enhanced viral infectivity and reduced interferon production are associated with high pathogenicity for influenza viruses
Source: PLoS Comput Biol. 2023 Feb 9;19(2):e1010886. doi: 10.1371/journal.pcbi.1010886 (PMC9946260; doi:10.1371/journal.pcbi.1010886)
Supplement: S3 Text — (DOCX) [file pcbi.1010886.s020.docx]

**Enhanced infectivity and attenuation of interferon production are associated with high pathogenicity for influenza viruses**

Ke Li, James M McCaw, Pengxing Cao

S3 Text

A simulation-estimation study

The purpose of the simulation and estimation study is to explore if extra macrophage data provides more information to better estimate model parameters and reproduce viral and macrophage dynamics. We use simulation and mathematical model to show that macrophage data can be used to accurately the recruitment rate of macrophages, inferring the timing and strength of the increase of macrophage during influenza viral infection. By contrast, viral load data alone cannot be used to reliably recover the macrophage dynamics. Hence, the combination of viral load and macrophage data in model fitting enhances our ability to replicate macrophage dynamics and allows us to explore detailed macrophage-virus interactions, e.g., the contribution of macrophages (both in timing and strength) on viral clearance.

## Generation of synthetic viral load and macrophage data

We first generate synthetic data for viral loads and macrophages mimicking the experimental procedure. We assume “true” parameter values are known (see Table 1). We do model (details in main text) simulation using the parameter values to get “true” trajectory of viral load and macrophages dynamics across infection period. The “true” parameters are selected such that (1) viral load peaks around day 2 post infection; (2) viral load is below a detection limit around day 7 post infection; (3) the adaptive immune responses (i.e., antibody and CD8+T cells) only activate after day 5 post infection; (4) viral infection can be suppressed timely when both arms of adaptive immune responses (i.e., antibody and CD8+T cells) are presented; (5) virus can be cleared but clearance delays when there is only an antibody response, and (6) a chronic infection occurs when an antibody repones is suppressed (Fig 1C). A detailed model dynamics see Fig 1.

Further, we get observation viral load and macrophage data from the “true” trajectory by adding lognormal noise and imposing a detection limit. Mathematically, the measured viral load $V_{n,\tau}$ and macrophage $M_{n,\tau}$ for each mouse $n=1, 2,\ldots, N$ and measuring time point $\tau=1, 2, ..., T$ are given by

$V_{n,\tau}=\left\{ \begin{aligned} V_{true}\left( \tau,\Phi\right){10}^{e_{n, \tau}}, \mathrm{if} V_{true}\left( \tau,\Phi\right){10}^{e_{n, \tau}}\geq\Theta\\ 0, & otherwise \end{aligned} \right.$ and $M_{n,\tau}=M_{true}\left( \tau,\Phi\right){10}^{e_{n, \tau}}$

$\Phi$ is a vector of “true” parameter values. $e_{n,\tau}$ is the measurement error, which follows $N(0,\sigma)$, and $\sigma=1$ for viral load data and $\sigma=0.1$ for macrophage data. $\Theta$ is detection limit. $V_{true}\left( \tau,\Phi\right)$ is the “true” viral load value at each measuring time $\tau$, and $M_{true}\left( \tau,\Phi\right)$ is the “true” macrophage value at each measuring time $\tau$. Here, we select $N=5$ to indicate at each measuring time 5 data points are measured, and we set $\tau=7$. As shown in Fig 1A and 1B, the open circles indicate measured data points at each measuring time for viral load and macrophages, respectively. The red cycles indicate the mean value of the 5 data point at each time, and we only use the red data points of viral load and macrophage populations for the model estimation.

**Fig 1.** The synthetic data for (A) viral load, (B) macrophages. (C) “true” parameter values are selected such that viral loads have different behaviours when different arms of adaptive immune responses are suppressed.

**Table 1.** “true” parameter values to generate true viral load and macrophages trajectories

| Parameter | Description | Value | Unit |
| --- | --- | --- | --- |
| $s_{M}$ | Recruitment rate of macrophages in the absence of infection | 3.3e+3 | /day |
| $k_{-1}$ | Conversion rate from $M_{1}$ to $M_{R}$ | 0.3 | /day |
| $k_{-2}$ | Conversion rate from $M_{2}$ to $M_{R}$ | 0.3 | /day |
| $\delta_{MA}$ | Decay rate of activated $M1$and $M2$ macrophages | 1.1e-2 | /day |
| $k_{1}$ | Conversion rate from $M_{R}$ to $M_{1}$ | 0.4 | /day |
| $k_{2}$ | Conversion rate from $M_{R}$ to $M_{2}$ | 4e-6 | /day |
| $V_{50}$ | Half saturation of viral load to activate $M_{1}$ macrophages. | 1e+7 | PFU/ml |
| $\alpha$ | Effectiveness of $M_{2}$ attenuates $M_{R}$ to $M_{1}$ | 1e-4 | /cell |
| $D_{50}$ | Half saturation of dead cells | 1e+6 | cell |
| $g_{T}$ | Regrowth rate of epithelium | 0.8 | /day |
| $T_{max}$ | The maximal epithelium cells | 7e+7 | cell |
| $\delta_{I}$ | Decay rate of infected cells | 2 | /day |
| $\delta_{V}$ | Decay rate of virus | 20 | /day |
| $\kappa_{F}$ | Clearance rate of infected cells by interferons | 3 | /(day [$\mu_{F}$])  *[$\mu_{F}$] is the unit for interferon |
| $\kappa_{E}$ | Clearance rate of infected cells by CD8+T cells | 8 | /day |
| $\kappa_{D}$ | Clearance rate of dead cells by $M_{1}$ macrophages | 8e-7 | /(day cell) |
| $\delta_{D}$ | Decay rate of dead cells | 2 | /day |
| $p_{I}$ | Viral production rate | 210 | pfu/(ml cell day) |
| $\delta_{F}$ | Decay rate of interferons | 2 | /day |
| $\phi$ | Conversion rate from $T$ to $R$ | 0.33 | /(day [$\mu_{F}$]) |
| $\xi_{R}$ | Conversion rate from $R$ to $T$ | 2.6 | /day |
| $\mathcal{l}$ | Eclipse phase | 4 | /day |
| $t_{E}$ | Half saturation time of CD8+ T cell response | 5 | day |
| $t_{A}$ | Half saturation time of antibody response | 5 | day |

## Bayesian statistical inference

Two scenarios were considered, one of which is using viral load data only, and the other is using both viral load and macrophage data. We applied a Bayesian inference method to fit the dynamic model (detailed in the main text) to the log-transformed kinetic data. In detail, we use the model to estimate 8 parameters, and the parameter space is denoted as $\Phi=\log_{10} (s_{V}, \beta,q_{FI},q_{FM},s_{M},\kappa_{A}, q^{'},V_{0})$. Upon model calibration, we fixed all other parameters their “true” values as shown in Table 1.

The prior distribution for the estimated model parameters is given in Table S2 in S2 Text. The distribution of the observed log-transformed viral load and/or macrophage data is assumed to be a normal distribution with a mean value given by the model simulation results and standard deviation (SD) parameter with prior distribution of a normal distribution with a mean of 0 and an SD of 1.

Model fitting was performed in R (version 4.0.2) and Stan (Rstan 2.21.0). Samples were drawn from the joint posterior distribution of the model parameters using Hamiltonian Monte Carlo (HMC) optimized by the No-U-Turn Sampler (NUTS) (details see Chatzilena et al. (2019)) [1]. In particular, we used 4 chains with different starting points and ran 8000 iterations (first 3000 samples are burn-in) for each chain when only viral load data is used. We also tried to run 2000, 4000 and 6000 iterations, respectively and effective sample size is small. When viral load data and macrophages are both used, we ran 4 chains with 2000 iterations (first 1000 samples are burn-in) for each chain.

## Predictive check

**Fig 2. Results of model fitting for virological and macrophage data.** Data are presented by solid circles. (A) shows a 95% prediction interval (shaded area) of reproduced viral dynamics by using viral load data only (green) or both viral load and macrophage data (pink). (B) shows a 95% prediction interval (shaded area) of reproduced macrophage kinetics by using viral load data only (green) or both viral load and macrophage data (pink).

## Posterior comparison

**Fig 3. Posterior distributions of estimated parameters.** Purple bars show posterior density of parameters when only viral load data is used. Green bars show posterior density of parameters when both viral load and macrophage data are used. Red lines indicate the “true” parameter values.

## Diagnostics

**Fig 4. Trace plots of estimated parameters using only viral load data.** Four chains were used with 8000 iterations and first 3000 iterations as burn-in (grey area). All parameters are log-transformed. The parameter vector is $\Phi=log10(s_{V}, \beta,q_{FI},q_{FM},s_{M},\kappa_{A}, q^{'},V_{0})$.

**Fig 5. Trace plots of estimated parameters using both viral load and macrophage data.** Four chains were used with 2000 iterations and first 1000 iterations as burn-in (grey area). All parameters are log-transformed. The parameter vector is $\Phi=\log10(s_{V}, \beta,q_{FI},q_{FM},s_{M},\kappa_{A}, q^{'},V_{0})$.

**Reference**

1. Chatzilena, A., van Leeuwen, E., Ratmann, O., Baguelin, M. and Demiris, N., 2019. Contemporary statistical inference for infectious disease models using Stan. *Epidemics*, *29*, p.100367.
